# Understanding the long-term interplay of SARS-CoV-2 immune and inflammatory responses with proteases in COVID-19 recovery: a longitudinal study

**DOI:** 10.3389/fimmu.2025.1517933

**Published:** 2025-06-10

**Authors:** Natalia Ćwilichowska-Puślecka, Aleksandra Makowiecka, Małgorzata Kalinka, Katarzyna Groborz, Tobiasz Puślecki, Marcin Drąg, Krzysztof Simon, Krystyna Dąbrowska, Monika Pazgan-Simon, Marcin Poręba

**Affiliations:** ^1^ 1Faculty of Chemistry, Wroclaw University of Science and Technology, Wroclaw, Poland; ^2^ Department of Systems and Computer Networks, Wroclaw University of Science and Technology, Wroclaw, Poland; ^3^ Department of Infectious Disease and Hepatology, Wroclaw Medical University, Wroclaw, Poland; ^4^ Department of Infectious Diseases, Regional Specialist Hospital, Wroclaw, Poland; ^5^ Faculty of Medicine, Wroclaw University of Science and Technology, Wroclaw, Poland

**Keywords:** COVID-19, immune response, mass cytometry (CyTOF), proteases, luminex (xMAP) method, SARS-CoV-2 infection, long COVID, matrix metalloproteinases

## Abstract

**Introduction:**

The immune and inflammatory responses following SARS-CoV-2 infection, particularly in the context of long COVID, remain critical areas of study. Understanding these responses is essential for addressing the long-term health impacts of COVID-19. Recent research also highlights the pivotal role of proteases in modulating immune responses and contributing to disease severity, making them a key focus of our analysis.

**Methods:**

We conducted a longitudinal analysis of 72 convalescent COVID-19 patients, assessing recovery at three key time points: immediately post-discharge, one month later, and three months post-infection. Additionally, a subset of 15 patients was followed up two years post-COVID-19. Clinical parameters, including demographics, comorbidities, treatment modalities, and COVID-19 severity, were evaluated. Using CyTOF technology, we characterized over 30 immune cell subsets, including granulocytes, T cells, B cells, NK cells, and monocytes. We also performed multiplexed analyses of blood samples to profile cytokines, chemokines, growth factors, proteases, and COVID-19-related proteins.

**Results:**

Our comprehensive approach revealed significant changes in the immune system over time, highlighting the role of specific immune cells and proteases in the recovery process. Key findings include a decreasing deregulatory effect on immune responses exerted by subsequent SARS-CoV-2 variants Alpha, Delta, and Omicron.

**Conclusion:**

This study provides an in-depth understanding of the molecular dynamics of immune recovery following COVID-19. By integrating clinical profiling, plasma multiplex analysis, antibody profiling, mass cytometry immunophenotyping, in vitro PBMC stimulation, and the role of proteases, we offer valuable insights into the complex interplay of immune, inflammatory, and protease-mediated responses in individuals recovering from COVID-19.

## Introduction

The COVID-19 pandemic, caused by SARS-CoV-2 (severe acute respiratory syndrome coronavirus 2), has sparked global research to unravel the virus-host interactions and immune responses (1). Since its emergence, COVID-19 has presented significant public health challenges, demanding detailed understanding of its complex nature. Clinical presentations of SARS-CoV-2 infection range from asymptomatic or mild upper respiratory symptoms to severe viral pneumonia and ARDS (acute respiratory distress syndrome) (2–4). The innate immune system is activated immediately by infected cells and viral RNA. Pattern-recognition receptors (e.g. TLRs, toll-like receptors) sense the virus and induce antiviral interferons and proinflammatory cytokines (5). Natural killer cells and macrophages respond to infected cells, while dendritic cells process antigen and stimulate adaptive immunity. Concurrently, T and B lymphocytes expand, generating virus-specific cellular immunity and neutralizing antibodies (6). In most individuals this response contains the infection. However, a subset of patients develop an aberrant hyperinflammatory response called cytokine storm that drives pathology (7). Severe COVID-19 is characterized by high levels of inflammatory mediators; including interleukins IL-2, IL-6, IL-7, IL-10, TNF-α (tumor necrosis factor alpha), and chemokines such as IP-10 (interferon γ-induced protein 10kDa) and MCP-1 (monocyte chemoattractant protein 1), together with profound lymphopenia​. These dysregulated responses are strongly associated with disease severity and organ damage. Indeed, uncontrolled cytokine release is a hallmark of severe COVID-19 and a leading cause of respiratory failure and mortality (8)​. For example, patients with critical illness show IL-6 and TNF-α levels many-fold higher than those in mild cases (9)​. Emerging evidence also implicates platelet activation, complement and coagulation cascades, and endothelial dysfunction in the pathogenesis of COVID-19 (10). Despite these advances, the precise determinants that distinguish recovery from chronic inflammation remain incompletely understood. In this context, the concept of immune plasticity is important: after acute infection, immune cell subsets can dynamically reprogram (e.g. memory T cell formation, regulatory cell expansion, or exhaustion of effector cells) to restore homeostasis.

Proteases and their inhibitors occupy central roles at the intersection of viral entry, inflammation, and tissue remodeling (11). Host proteases (TMPRSS2, furin, cathepsins) are key cofactors for SARS-CoV-2 cell invasion (12). Similarly, cellular peptidases such as DPPIV/CD26 (dipeptidyl peptidase IV) and NRP1 (neuropilin-1) can modulate infectivity and immune signaling (13). Beyond the virus life cycle, multiple proteolytic enzymes produced by immune cells shape the course of COVID-19 and recovery. For instance, neutrophil elastase (NE) degrades epithelial cadherin and surfactant proteins, disrupting lung barriers and altering cytokine activity​. Under normal conditions, NE is tightly controlled by endogenous inhibitors like α1-antitrypsin, but in severe inflammation this balance is lost (14). Moreover, cytotoxic lymphocytes release granzymes which, while inducing apoptosis of infected cells, can also persist extracellularly, cleaving extracellular matrix (ECM) proteins and activating pro-inflammatory cytokines​. For example, granzyme B can cleave fibronectin and laminin and liberate matrix-bound growth factors, propagating inflammation; elevated granzyme B levels have been observed in chronic COVID-19 and correlate with tissue damage ​ (15). Finally, the matrix metalloproteinases (MMPs) are zinc-dependent endopeptidases that remodel the ECM and regulate cytokine availability. MMPs like MMP-9 and MMP-12 can activate latent cytokines (TNF-α, IL-1β) and degrade extracellular proteins (collagens, elastin, basement membranes) to facilitate cell migration and repair​. Dysregulated MMP activity is linked to lung injury and fibrosis in viral infections (16). The interplay between proteases and inhibitors thus embodies immune plasticity; it reflects how the immune system dynamically shifts between pro-inflammatory and resolution phases. Tracking these proteins in blood might provide insight into ongoing inflammation versus healing processes.

The phenomenon of long COVID (post-acute sequelae of SARS-CoV-2 infection) underscores the need to understand extended immune and inflammatory responses (17). Long COVID refers to persistent or relapsing symptoms lasting weeks to months after the acute phase​. While most patients eventually recover, a substantial fraction (~10–20%) develop prolonged effects such as fatigue, dyspnea, cognitive “brain fog,” and other systemic complaints (18). These chronic manifestations can last for many months and often wax and wane. This condition poses a major public health burden. Thus, elucidating the biological underpinnings of sustained inflammation and immune dysregulation is a critical priority, both for preventing chronic disease and for guiding future therapies. In this longitudinal study of COVID-19 convalescent patients, we leverage these insights to comprehensively profile post-infection recovery. We followed 72 individuals through early convalescence (hospital discharge) and up to three months post-infection (with a subset at two years). Clinical data (demographics, comorbidities, acute disease severity) were correlated with high-dimensional immunological readouts. We performed multiplex assays on blood plasma to quantify anti-SARS-CoV-2 antibody titers, a broad panel of cytokines, chemokines, growth factors, proteases, and protease inhibitors. In parallel, we employed mass cytometry (CyTOF, cytometry by time-of-flight) to enumerate and phenotype over 30 leukocyte subsets (granulocytes, T cells, B cells, NK cells, monocytes, among others). This integrative approach allows us to link molecular markers (e.g. MMPs, TIMPs – tissue inhibitors of metalloproteinases) with cellular immune dynamics and clinical outcomes. Our goals are to define the trajectory of immune restoration after COVID-19, identify biomarkers of persistent inflammation or immune dysregulation, and understand how perturbations in proteolytic pathways may contribute to long-term sequelae. By combining plasma multiplexing and deep immune profiling, we aim to uncover mechanisms that could guide future therapies and public health strategies for mitigating the enduring impacts of COVID-19.

## Materials and methods

### Patients cohort and clinical data collection

This study included 72 COVID-19 patients, either hospitalized or treated at the Department of Infectious Disease and Hepatology, Wroclaw Medical University, Poland. Ethical approval was granted by the Silesian Medical Community of Wroclaw (KB/242/2020), and all participants provided written informed consent. Patients were monitored for three months post-COVID-19 convalescence, with some examined again after two years. Inclusion occurred at hospital discharge. Clinical data collected during hospitalization included lung ultrasound, blood pressure, age, gender, BMI (body mass index), and WHO (The World Health Organization) disease severity classification. Severity was categorized as mild (symptoms without pneumonia), moderate (pneumonia without oxygen), severe (pneumonia requiring oxygen), or critical (requiring intensive care due to complications like ARDS or septic shock). Additional data included demographics, comorbidities, treatment modalities, and pneumonia presence.

### Blood sample collection and PBMC/plasma isolation

Peripheral blood mononuclear cells (PBMCs) and plasma were collected from COVID-19 convalescent individuals at five key time points: at discharge (t0), one month (t1), three months (t3), post-vaccination (tv), and two years post-discharge (tx or 2-years). For each patient, 2x10 mL of blood was collected in EDTA tubes and processed within 4 hours. Each 10 mL sample was diluted with 10 mL of 2% fetal bovine serum, FBS (Gibco, A5256801) in phosphate-buffered saline, PBS (Sigma-Aldrich, D8537) and layered on 10 mL of Lymphoprep (Serumwerk Bernburg AG, 1858). The samples were centrifuged at 300 x g for 30 minutes to separate plasma and PBMCs. Plasma was collected, aliquoted into 1 mL tubes, and stored at -80°C. The PBMC layer was transferred to a new tube, washed with 2% FBS in PBS, and residual red blood cells were lysed using RBC (Biolegend, 420302) lysis buffer. The PBMCs were washed again, counted, and cryopreserved in 10% DMSO in FBS, then stored in liquid nitrogen for long-term preservation.

### The analysis of antibodies and proteins level in plasma

The concentrations of anti-SARS-CoV-2 antibodies and selected proteins in plasma samples from COVID-19 convalescent individuals were analyzed using xMAP^®^ Luminex technology with dedicated assay kits (**Supplementary Table S1**). All assays were performed according to the manufacturer’s instructions. Before testing, samples were completely thawed, vortexed, and centrifuged at 1000 × g for 5 minutes to remove particulates. The washing solution, Sheath Fluid Plus, concentrated human recombinant standards, and quality controls were provided by the manufacturer. A broad range of standards was used to establish standard curves. Standards, quality controls, background controls, and patient plasma samples were incubated with pre-mixed microbeads while shaking, following the manufacturer’s protocols. After incubation, the samples were washed and re-incubated with detection antibodies. Following another wash step, samples were further incubated with Streptavidin-Phycoerythrin. The plates were then washed three times, and the samples were resuspended in Sheath Fluid Plus. Analyte levels were detected using the Luminex^®^ 200™ system (100 µL volume, 50 beads per bead set). Analytes levels were expressed as median fluorescence intensity (MFI). The fluorescence signals from samples and standards were detected using the Luminex 200 system and automatically processed using the corresponding software. Sample fluorescence values were fitted to the standard curves using Belysa Immunoassay Curve Fitting Software to calculate final concentrations. Data were normalized as necessary, and statistical analyses were conducted using GraphPad Prism 10 software.

### PBMC stimulation

For PBMC stimulation, peptides and proteins from JPT Peptide Technologies were used (JPT, 54406). Pepmix HIV-1 (GAG, PM-HIV-GAG PepMix™) Ultra was applied as an HIV control, while Pepmix SARS-CoV-2 (Spike glycoprotein, PM-WCPV-S-1 PepMix™) was used for COVID-19 stimulation. The complete media consisted of RPMI 1640 (Sigma Aldrich, R8758) with 2 mM L-Glutamine (Gibco, 25030-081) and 10% heat-inactivated fetal calf serum (Sigma-Aldrich, N4762). DMSO diluted 1:200 in media served as the negative control. Stock peptide solutions were prepared by dissolving 25 μg of peptides in 50 μL DMSO and diluted 1:200 in media. For the assay, 80 μL of peptide solutions and controls were added to a 96-well plate, followed by 80 μL of cell suspension at 2.5 x 10^6^ cells/mL. PBMCs were incubated for 12 hours at 37°C with 5% CO_2_. After incubation, cells were centrifuged, and the supernatant was collected for further analysis using Luminex xMAP technology.

### Preparation of metal-conjugated antibodies

Monoclonal antibodies targeting selected proteins were conjugated with specific metal isotopes using the Maxpar^®^ X8 Antibody Labeling Kit (Standard Biotools, 201300), following the detailed protocol described by Han et al. (19) (**Supplementary Table S2**). Metals were selected to serve as complementary markers for the Maxpar^®^ Direct™ Immune Profiling Assay™. Briefly, the selected metal isotope (as chloride salt, MeCl_3_) was initially loaded onto the Maxpar^®^ X8 polymer. Subsequently, 100 μg of each monoclonal antibody was reduced using TCEP (Sigma-Aldrich, C4706) and then incubated with the metal-loaded polymer. After incubation, the concentration of the metal-tagged antibody was measured at A280 nm. The conjugate was then diluted to a concentration of 0.5 mg/mL using Antibody Stabilizer (Sigma-Aldrich, 55514) and stored at +4°C until use.

### Immune profiling and protein detection by mass cytometry

Whole blood samples (10 mL) were collected in EDTA tubes and processed within 4 hours. A 373 μL aliquot of whole blood was added to a Maxpar^®^ Direct™ Immune Profiling Assay™ (Standard Biotools, 201325) tube (**Supplementary Table S3**), followed by in-house metal-conjugated antibodies (1 μL of 0.5 mg/mL per sample). After a 30-minute incubation at 37°C, red blood cells were lysed using 3 mL of 1x lysis buffer with a 7-minute incubation in the dark. Cells were centrifuged for 5 minutes at 300 x g between steps. Next, the cells were washed with 1 mL of PBS and incubated with lysis buffer for 10 minutes. After three washes with Cell Staining Buffer, CSB (Standard Biotools, 201068), the cells were fixed for 10 minutes with 1.6% formaldehyde (Thermo Scientific, 28908), followed by centrifugation at 900 x g. The cell pellet was resuspended in Fix and Perm Buffer (Standard Biotools, S00092), centrifuged, and incubated overnight at 4°C with Ir intercalator (Standard Biotools, S00093) (0.125 μM). Samples were either immediately measured or stored at -80°C. Before mass cytometry, cells were washed twice with Cell Staining Buffer and Cell Acquisition Solution, CAS (Standard BioTools, 201240), diluted in EQ Four Element Calibration Beads (Standard Biotools, 201078) (10%) to 10^6^ cells, and analyzed with a Helios mass cytometer. Approximately 0.5x10^6^ cells were recorded per sample at 300-400 events per second. Data were normalized using the Normalizer v0.3 from Nolan Lab (20) - www.github.com/nolanlab. Data analysis was performed with Cytobank (21) - www.premium.cytobank.org.

### Data visualization and statistical analysis

The statistical analysis and data visualization was performed using GraphPad Prism 10, Cytobank software and Biorender. For hierarchical clustering and heat-map generation, we used R software. Graphs were generated in GraphPad Prism 9. Normally distributed data were compared by two-tailed Student’s t-tests (unpaired for independent samples, paired for matched measures), while non-normal data were analyzed by the Mann-Whitney U test. Statistical significance is annotated as ns (p > 0.05), * (p ≤ 0.05), ** (p ≤ 0.01), *** (p ≤ 0.001) or **** (p ≤ 0.0001).

## Results

### Insights from the clinical data of COVID-19 convalescent individuals

**Figure 1:**
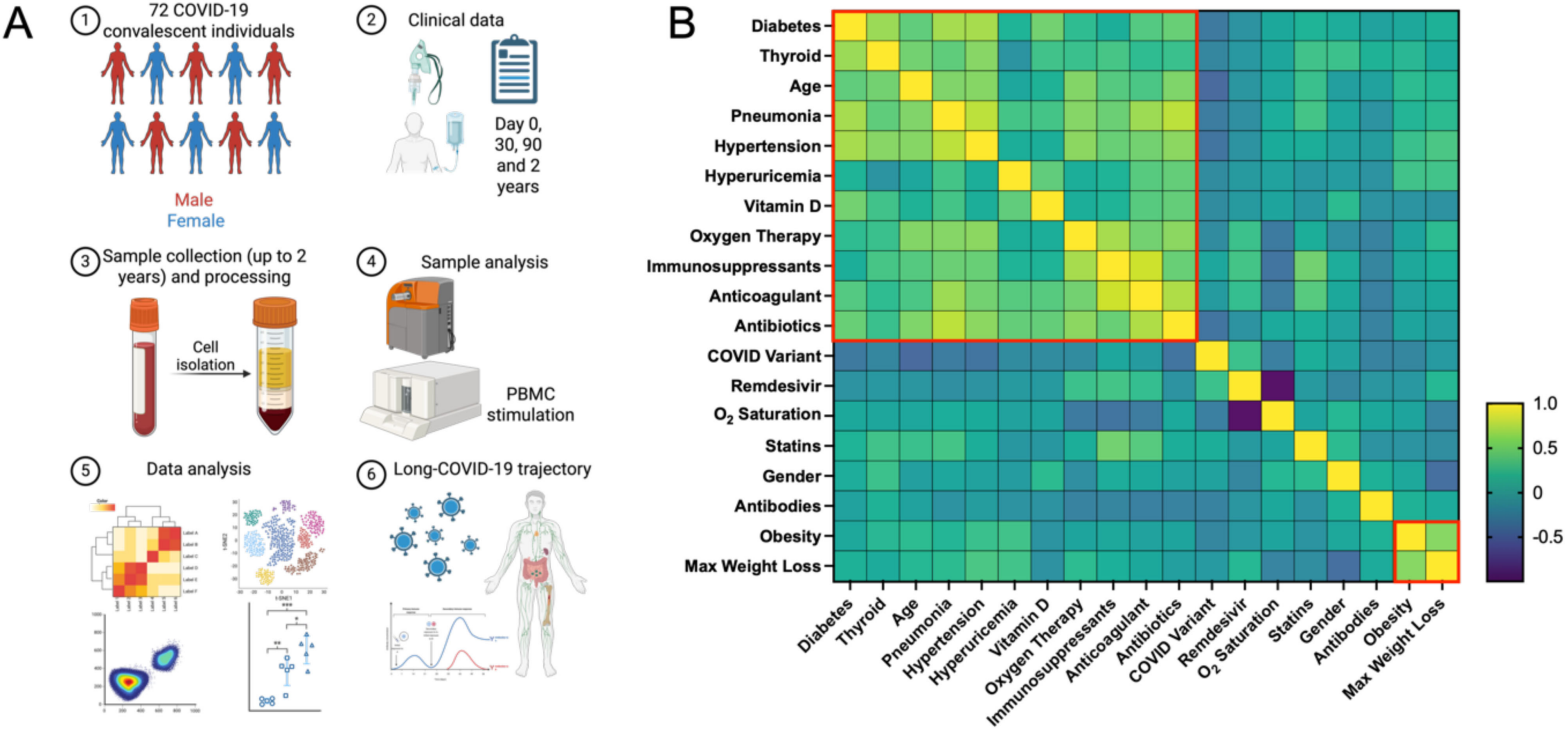
The analysis of COVID-19 convalescent patients with multimodal approach. **(A)** Schematic outline of the methodology applied for comprehensive profiling of post-COVID immune system dynamics in convalescent patients. The study involved recruiting 72 COVID-19 patients and analyzing blood samples using Luminex assays, mass cytometry, and PBMC stimulation. Research data were collected at multiple time points and integrated with clinical data recorded during hospitalization. **(B)** The heatmap visualizes the correlation matrix of various clinical and demographic factors of COVID-19 convalescent patients. Variables include age, sex, BMI, COVID-19 variant (estimated based on date), duration of hospitalization, oxygen therapy, WHO clinical scores at different time points, presence of pneumonia, lung ultrasound findings at discharge and one month later, comorbidities, COVID-19 therapies (such as Vitamin D supplementation or Remdesivir), and EQ-5D-5L health outcomes (mobility and pain/discomfort). Hierarchical clustering was applied to group variables based on their correlation coefficients. Dendrograms are shown on both the top and left sides of the heatmap, illustrating hierarchical relationships. Red boxes highlight clusters formed by variables with strong positive correlations.

This study examines a cohort of 72 convalescent COVID-19 patients (**Table 1**), comprehensively analyzed with various methodologies at three key points: immediately post-discharge (t0), one month later (t1), and three months post-infection (t3) (**Figure 1A**). Additionally, 15 patients were followed two years post-discharge. The cohort, with an average age of 56 years, consisted of 39 females and 33 males, with a majority classified as overweight or obese. The clinical profiles highlighted varying COVID-19 severity, comorbidities (such as diabetes, hypertension, and obesity), and treatment modalities, including antibiotics and immunosuppressants (**Figure 1B**). Differences between severe and mild cases, classified per WHO guidelines, were significant. Severe cases typically involved pneumonia, respiratory distress, or oxygen therapy, while mild cases did not. Older patients (median age 65 vs. 54 years), with higher BMIs (median 32 vs. 26), had longer hospital stays (15 vs. 2 days). Notably, 85% of severe cases required oxygen therapy, compared to 20% of mild cases. Health-related quality of life (HRQoL), measured via EQ-5D-5L (a standardized measure of health-related quality of life developed by the EuroQol Group), was significantly impacted in severe cases, with higher reports of mobility issues, pain, and psychological distress. The study also noted a higher incidence of hypertension, diabetes, and obesity among severe cases, underscoring the influence of these comorbidities on disease progression. Therapeutic interventions were shaped by the timing of infection and clinical status upon hospital admission. Lung ultrasounds revealed more abnormalities in severe cases (80% showing signs of pneumonia and complications) compared to 25% of mild cases.

**Table 1:** Clinical and demographic characteristics of COVID-19 patients (n = 72).

Variable	Statistic	Values
Age (years)	Range	23 to 90
	Median	59
	IQR*	21
Gender	Male (%)	45% (33/72)
	Female (%)	55% (40/72)
Body Mass Index (BMI)	Range	18.56 to 46
	Median	28
	IQR*	6.61
Hospitalization status	Never hospitalized (%)	59% (43/72)
	Hospitalized (%)	41% (30/72)
Oxygen therapy	Yes (%)	27% (20/72)
	No (%)	73% (53/72)
Health outcomes (EQ-5D-5L)	Mobility (median IQR*)	2 1
	Self-Care (median IQR*)	1 0
	Usual Activities (median IQR*)	1 0
	Pain/Discomfort (median IQR*)	2 0
	Anxiety/Depression (median IQR*)	1 0
Health scale (1-100)	Range	2 to 100
	Median	90
	IQR*	15
COVID-19 therapy	Antiviral therapy (%)	15% (11/72)
	Steroid therapy (%)	20% (15/72)
	Other therapies (%)	10% (7/72)
Comorbidities	Hypertension (%)	30% (22/72)
	Diabetes (%)	15% (11/72)
	Cardiovascular disease (%)	10% (7/72)
	Respiratory disease (%)	5% (4/72)
	Other comorbidities (%)	25% (18/72)
Pneumonia severity	Yes (%)	30% (22/72)
	No (%)	70% (51/72)
Ultrasound at discharge	Range	1 to 6
	Median	1
	IQR*	1
Ultrasound one month later	Range	0 to 6
	Median	1
	IQR*	1
COVID-19 variant	Alpha (%)	31,5% (23/72)
	Delta (%)	44% (32/72)
	Omicron (%)	23% (17/72)
	Unknown (%)	1,5% (1/72)
IQR* - Interquartile Range		

Table presents a summary of key clinical and demographic characteristics of 72 COVID-19 patients. It includes data on age, gender, body mass index (BMI), hospitalization status, oxygen therapy usage, and health outcomes measured by the EQ-5D-5L scale. The table also details COVID-19 therapy types, the prevalence of comorbidities, pneumonia severity, and lung ultrasound results at discharge and one month later. Additionally, it provides information on the distribution of COVID-19 variants among the patients. IQR is an interquartile range.

When analyzing the cohort based on COVID-19 variants, patients were grouped by the original strain (March-June 2020), Alpha (October 2020-February 2021), Delta (April-August 2021), and Omicron (since December 2021). The Alpha and Delta variants were linked to more severe outcomes, including longer hospital stays and higher oxygen therapy needs. A strong correlation was found between severe COVID-19 and comorbidities such as hypertension, diabetes, and obesity. Treatments like anticoagulants and antibiotics were more commonly used in severe cases to manage serious symptoms. These findings emphasize the importance of early identification and management of high-risk patients, particularly older individuals, to mitigate disease severity and improve outcomes.

The observations align with established research. Numerous studies consistently show that individuals with comorbidities such as hypertension, diabetes, and obesity are at higher risk for severe COVID-19 (22, 23). The use of anticoagulants in severe cases is well-documented, given the increased risk of thromboembolic events (24). Similarly, antibiotics are commonly used to prevent secondary bacterial infections, as recommended by clinical guidelines (WHO/2019-nCoV/clinical/2020.5). Oxygen therapy remains a critical intervention for severe COVID-19, as supported by WHO and CDC (Centers of Disease Control and Prevention) recommendations for managing respiratory distress in severe cases. The impact of severe COVID-19 on quality of life, particularly regarding physical mobility, pain, and mental health, is well-documented (25, 26). Large cohort studies and meta-analyses show that older adults face greater risks of severe symptoms and prolonged recovery, underscoring the need for targeted interventions to improve outcomes in high-risk patients (27, 28).

### Longitudinal patterns of anti-SARS-CoV-2 antibodies following recovery and vaccination of COVID-19 convalescent individuals

**Figure 2:**
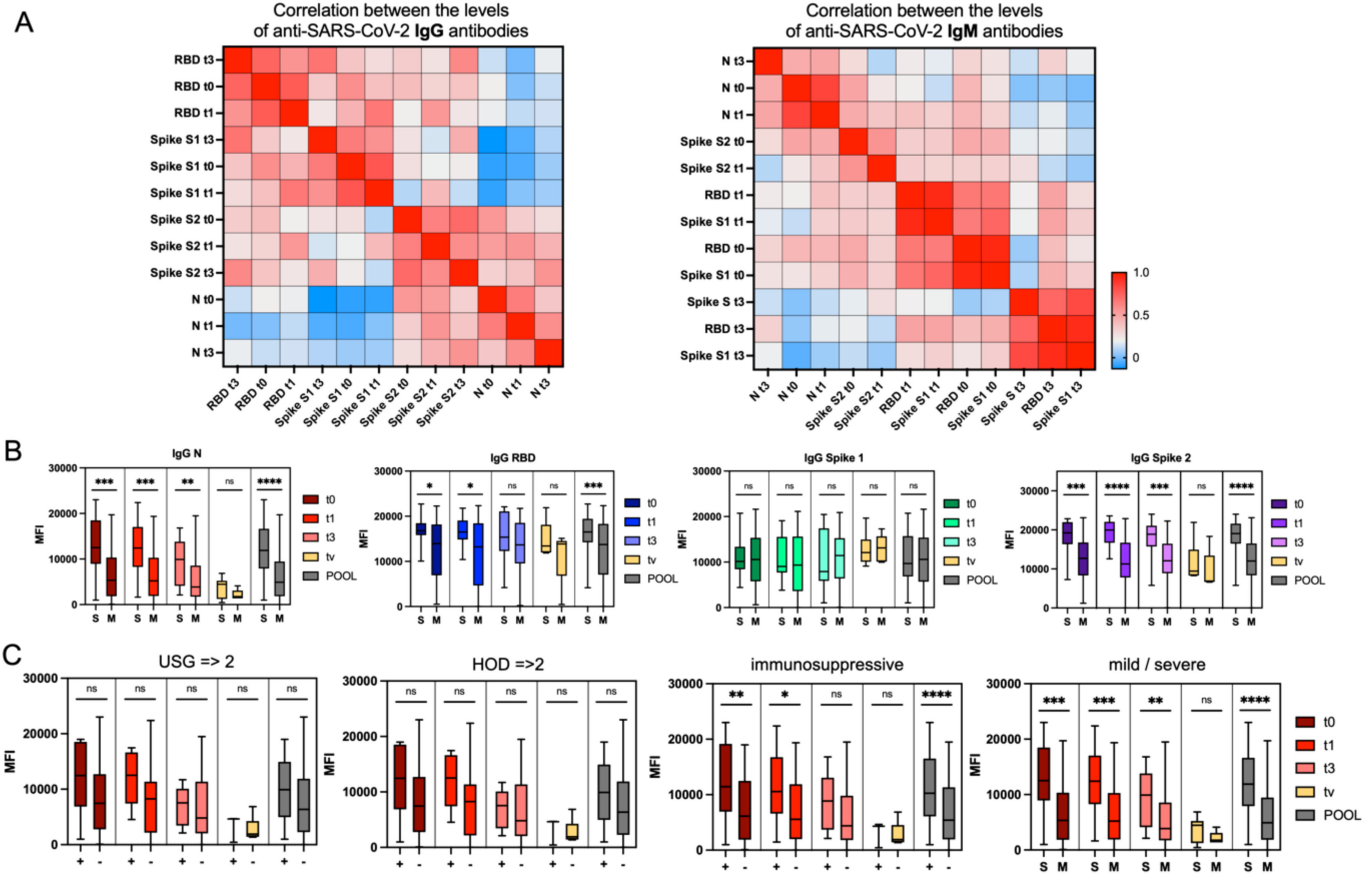
Time-related patterns of anti-SARS-CoV-2 antibodies. **(A)** Heatmaps showing hierarchical clustering of correlations between the levels of anti-SARS-CoV-2 IgG (left) and IgM (right) antibodies measured at four time points: at hospital discharge (t0), 30 days post-discharge (t1), 90 days post-discharge (t3), and post-vaccination (tv), as well as pooled data. The color-coded legend indicates correlation strength expressed as the Pearson correlation coefficient, with red representing the highest positive correlation. **(B)** Trajectory of anti-SARS-CoV-2 antibody levels over time in two groups of COVID-19 convalescent patients: those who experienced a mild (M) course and those with a severe (S) course of the disease. Graphs present antibody titers expressed as relative mean fluorescence intensity (MFI) measured by xMAP^®^ Luminex. Statistical significance of differences between the M and S groups is indicated. **(C)** Trajectory of anti-SARS-CoV-2 antibody levels against the nucleocapsid IgN protein at the four time points (t0, t1, t3, tv) and pooled, stratified by lung ultrasound results (positive [+] for confirmed changes; negative [–] for no changes), number of comorbidities (2 or 3 comorbidities [+] vs. 0 or 1 [–]), use of immunosuppressive treatment, and severity of the disease. Statistical significance of differences between the groups is indicated. Statistical significance is annotated as: ns (p > 0.05), * (p ≤ 0.05), ** (p ≤ 0.01), *** (p ≤ 0.001) or **** (p ≤ 0.0001).

The course of COVID-19 and the immune response to SARS-CoV-2 infection have been studied intensively since the pandemic’s onset. One critical aspect of this response is antibody production, which plays a key role in neutralizing the virus and providing immunity (29). In this study, we measured various SARS-CoV-2-specific antibodies in convalescent patients to understand immune response dynamics during recovery and post-vaccination. The antibodies measured included those against the nucleocapsid (N) and spike (S) proteins, with a focus on IgG and IgM targeting the spike protein’s receptor-binding domain (RBD) and subunits (S1, S2), as these are crucial for immunity. We conducted this study at four time points: immediately after recovery (t0), one month later (t1), three months post-recovery (t3), and post-vaccination (tv). IgG RBD and IgG Spike S1 antibodies showed strong correlations across time points, indicating a consistent immune response. For example, IgG RBD at t0 was highly correlated with levels at t1 (r = 0.77) and t3 (r = 0.74), and similarly, IgG Spike S1 at t0 correlated with levels at t1 (r = 0.78), demonstrating stable antibody responses over time. (**Figure 2A**). Our analysis revealed a general decline in antibody levels from t0 to t3, within three months post-infection (**Figure 2B**, **Supplementary Figures S1, S2**). Specifically, IgG antibodies (IgG N, IgG RBD, and IgG Spike S2) showed a steady decrease. A similar trend was observed for IgM antibodies, with reductions in levels of IgM N, IgM RBD, IgM Spike S1, and IgM Spike S2.

We grouped patients into several categories to identify clinical factors that could influence antibody levels: (1) COVID-19 variant, (2) mild vs. severe disease, (3) male vs. female, (4) HOD (Hypertension, Obesity, Diabetes) score, with HOD ≥2 indicating two or more comorbidities, (5) immunosuppressive treatment during hospitalization, and (6) pneumonia indicated by lung ultrasound. Patients with a mild course of COVID-19 showed lower levels of IgG N antibodies regardless of the time point. Those without immunosuppressive treatment or with normal lung ultrasound results also had lower IgG N levels (**Figure 2C**). Higher levels of IgG N antibodies at discharge (t0) were moderately correlated with longer hospital stays (r = 0.48) and the need for oxygen therapy (r = 0.42), suggesting that patients with severe disease developed stronger antibody responses early on. This trend persisted at one month post-discharge (t1), with IgG N levels correlating with hospitalization duration (r = 0.41) and pneumonia (r = 0.39). Patients with pneumonia had higher IgG N levels and longer hospitalizations, linking severe disease to more robust antibody responses. These findings are consistent with existing research showing that severe cases of COVID-19, particularly those involving extended hospital stays and complications like pneumonia, elicit stronger and longer-lasting antibody responses. Studies have also shown that patients with severe COVID-19 tend to have higher levels of antibodies, particularly IgG, which are important for long-term immunity (30, 31).

### The analysis of cytokine, chemokine and growth factor in COVID-19 convalescent patients

**Figure 3:**
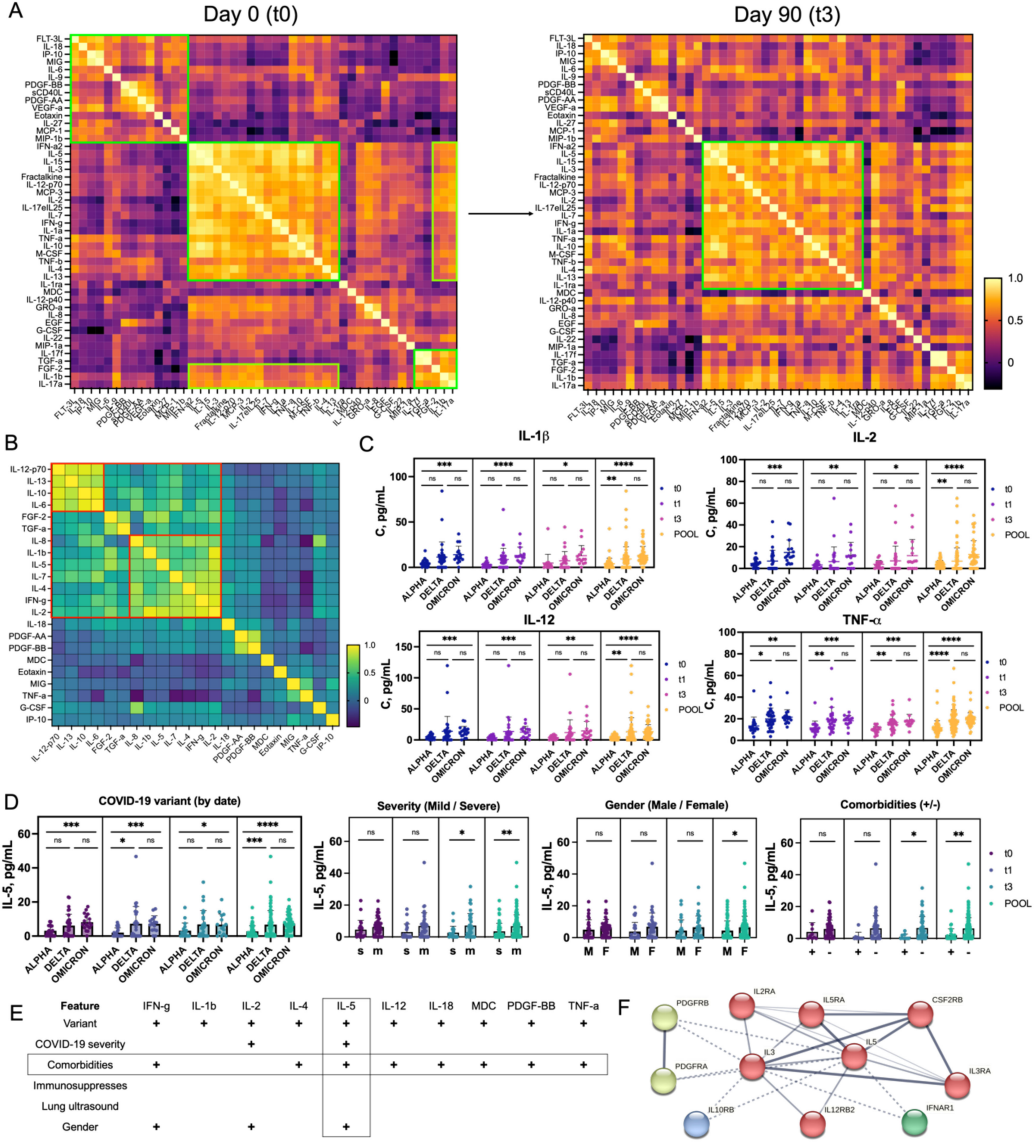
Cytokine, Chemokine, and Growth Factor Profile in COVID-19 Convalescent Individuals **(A)** Heatmaps representing the trajectory of changes in the broad immune landscape of 21 patients over 90 days post-recovery. Pearson correlation coefficients were calculated for each pair of immune parameters and visualized as heatmaps, where yellow indicates the highest correlation. Several coordinated immune response clusters were identified at t0, while partial dispersion of these clusters at t3 reflects recovery dynamics. **(B)** Heatmap representing the aggregated correlation (t0–t3) between selected immune proteins that showed significant differences between patient cohorts. Two distinct clusters were identified, each containing proteins with a Pearson correlation coefficient above 0.75. **(C)** Profiles of plasma concentrations of selected cytokines (IL-1β, IL-2, IL-12, TNF-α), showing statistically significant differences between patient groups stratified by COVID-19 variant (Alpha, Delta, Omicron) over the recovery period (t0 to t3) and in pooled data. The data demonstrate the highest cytokine concentrations in patients infected with the Omicron variant and the lowest for Alpha. **(D)** Differences in IL-5 concentrations across patient groups stratified by clinical features (COVID-19 variant, disease severity, gender, and presence of comorbidities) over time (t0 to t3 and pooled). Statistical significance is indicated. **(E)** Table showing proteins with statistically significant differences (p < 0.05) among various patient groups. Features showing the highest number of positive correlations with other factors are highlighted. IL-5 exhibits the most pronounced differences in concentration among groups. **(F)** STRING interaction network for IL-5, showing its closest molecular partners, organized into four clusters (red, green, blue, and yellow) arranged from the closest (red) to the most distant (yellow) interactions. Statistical significance is annotated as: ns (p > 0.05), * (p ≤ 0.05), ** (p ≤ 0.01), *** (p ≤ 0.001) or **** (p ≤ 0.0001).

We conducted an initial screening in 21 representative patients to identify key immunological indicators for more comprehensive analysis in the larger cohort of 72 COVID-19 convalescent individuals. xMAP was used to analyze plasma samples for cytokines, chemokines, and growth factors. The cytokines analyzed included IFNγ, IFNα2, IL-1α, IL-1β, IL-1RA, IL-2, IL-3, IL-4, IL-5, IL-6, IL-7, IL-8/CXCL8, IL-9, IL-10, IL-12 (p40), IL-12 (p70), IL-13, IL-15, IL-17A/CTLA8, IL-17E/IL-25, IL-17F, IL-18, IL-22, IL-27, TNFα, and TNFβ/Lymphotoxin-α (LTA). Chemokines included IP-10/CXCL10, MCP-1/CCL2, MCP-3/CCL7 (monocyte chemoattractant protein 3), MIG/CXCL9 (monokine induced by interferon-γ), MIP-1α/CCL3 (macrophage inflammatory protein-1α), MIP-1β/CCL4, MDC/CCL22 (macrophage-derived cytokine), GROα, RANTES/CCL5, Fractalkine/CX3CL1, and Eotaxin/CCL11. Growth factors such as EGF (epidermal growth factor), FGF-2/FGF-basic (fibroblast growth factor-2), Flt3 Ligand, G-CSF (granulocyte colony stimulating factor), M-CSF (macrophage colony stimulating factor), GM-CSF, PDGF-AA (platelet derived growth factor AA), PDGF-AB/BB, VEGF-A (vascular endothelial growth factor A), and TGFα (transforming growth factor α) were measured along with sCD40L. Hierarchical clustering and correlation analysis of protein levels from t0 to t3 revealed significant changes in the immune response of COVID-19 convalescent individuals over 90 days (**Figure 3A**). Initially, the immune response focused on combating the infection, evidenced by strong correlations among inflammatory cytokines like IL-1β, IL-6, IL-8, TNFα, and IFNγ. As recovery progressed, the emphasis shifted towards controlling inflammation and promoting tissue repair, highlighted by clustering of growth factors like VEGF-A, FGF, and PDGF. This transition from inflammation to repair is crucial for understanding the recovery trajectory in COVID-19 patients. At discharge, proteins such as chemokines IP-10 and MIG and growth factor FLT-3L showed high correlations, indicating a coordinated upregulation in response to COVID-19. Cytokines like IL-6, which have both pro- and anti-inflammatory effects (32), showed moderate correlations with multiple proteins, highlighting significant immune responses. By 90 days, weakening correlations between IP-10 and MIG suggested a return to baseline function, while new links between IL-17F and TGFα pointed to long-term immune regulation. Early recovery (t0) showed high pro-inflammatory activity, while late recovery (t3, 90 days) reflected ongoing adaptation and tissue repair.

To explore this further, we screened selected proteins in all 72 patients in our cohort. The proteins measured included chemokines and growth factors (Eotaxin, FGF-2, G-CSF, IP-10, MDC, MIG, PDGF-AA, and PDGF-BB), cytokines (IFNγ, IL-1β, IL-2, IL-4, IL-5, IL-6, IL-7, IL-8, IL-10, IL-12-p70, IL-13, IL-18, TNFα), and growth factor TGFα. Elevated levels of cytokines such as IL-6, IL-8, and TNFα have been linked to severe COVID-19 and the cytokine storm phenomenon, leading to ARDS and multi-organ failure (8). The role of chemokines like IP-10 and MIG in recruiting immune cells to infection sites and their elevated levels in severe cases has also been documented (8). Growth factors like FGF-2 and PDGF-BB are involved in tissue repair and fibrosis, crucial during recovery post-infection (33). Dynamic changes in protein levels post-discharge and post-vaccination provide insights into the long-term immune response and potential complications in convalescent patients (**Supplementary Figure S3A**). At discharge (t0), strong correlations among inflammatory cytokines like IL-1β, IL-2, IL-4, and IFNγ indicated a highly activated immune response. One month post-discharge (t1), proteins such as IL-6 and IL-13, linked to ongoing immune modulation, showed increased correlations, reflecting responses to persistent inflammation. By three months (t3), correlations among IL-6, IL-10, and other inflammatory markers indicated a shift towards resolving inflammation but with ongoing immune activity. An aggregated hierarchical clustering from t0 to t3 and principal component analysis (PCA) analysis identified two evident clusters: one with IL-12p70, IL-13, IL-10, and IL-6, and another with IL-8, IL-1β, IL-5, IL-7, IL-4, IFNγ, and IL-2 (**Figure 3B**, **Supplementary Figure S3B**). The first cluster is associated with immune modulation, with IL-12p70 and IL-6 driving initial immune responses, and IL-13 and IL-10 suppressing inflammation and promoting tissue repair (34). The second cluster contributes to both immediate and long-term immune responses, with IL-8 and IL-1β driving acute inflammation, and IL-5, IL-4, and IL-7 supporting immune regulation (34). IFNγ and IL-2 are essential for T cell responses and overall immune activation (35). Impaired production of IFNγ has been linked to COVID-19 severity, leading to prolonged inflammation and excessive production of pro-inflammatory cytokines (36). This pattern aligns with the literature, where a mix of pro-inflammatory and regulatory cytokines ensures an effective immune response while preventing excessive inflammation. Further subgroup analysis based on COVID-19 variant, disease severity, comorbidities, gender, immunosuppressive treatment, and lung ultrasound revealed significant findings (**Supplementary Figures S4–9**). Analysis of protein levels across Alpha, Delta, and Omicron variants showed significant temporal and variant-specific trends. IL-2, IL-4, IL-8, IL-5, IL-7, IFNγ, and TNFα demonstrated differences across these groups (**Figure 3C**, **Supplementary Figure S4**). Delta and Omicron patients exhibited prolonged inflammatory responses compared to Alpha patients, who showed a resolution of inflammation over time. Patients with varying pneumonia severity also showed differences, with higher IL-5 levels in convalescent patients with mild disease, aligning with reports that cytokine dysregulation varies widely among patients (**Supplementary Figure S5**) (37). In contrast to other inflammatory markers, IL-5 showed an opposite pattern: convalescent patients with mild disease had higher concentrations of IL-5 in their blood compared to individuals with severe disease (38). These observations align with studies indicating that cytokine dysregulation varies widely among patients. Additionally, patients with multiple comorbidities had elevated levels of cytokines like IFNγ, IL-2, IL-4, IL-5, IL-6, and IL-10, as well as growth factors like FGF-2, PDGF-AA, and PDGF-BB (**Supplementary Figure S6**). Patients receiving immunosuppressive treatment showed no significant differences in cytokine levels compared to those who did not receive treatment, indicating a return to immune profiles similar to mild patients (**Supplementary Figure S7**). No significant differences in protein levels were observed between patients with normal versus abnormal lung ultrasound results (**Supplementary Figure S8**). Gender analysis revealed higher levels of IFNγ, IL-2, and IL-5 in female patients (**Supplementary Figure S9**). IL-5 concentrations were notably lower in patients with mild disease and no comorbidities, suggesting it might serve as a positive prognostic marker for faster recovery (**Figures 3D, E**). IL-5 not only activates eosinophils but also ILC2 cells, B cells, Tc cells, and basophils. Its role in activating B1 B cells is particularly relevant for rapid antibody responses in the lungs. Similar IL-5-driven immune responses are seen in other respiratory viruses, such as influenza and RSV (39). The STRING (Search Tool for the Retrieval of Interacting Genes/Proteins) interaction map showed IL-5’s interactions with IL-2, IL-3, IL-12, and IL-10, proteins involved in immune regulation (**Figure 3F**) (40). These proteins are primarily involved in the regulation and modulation of the immune response. The low levels of IL-5 suggest a reduced Th2-mediated immune response and less eosinophilic inflammation, potentially leading to better outcomes in COVID-19 patients. IL-5’s role in immune regulation can be further studied, particularly its interactions with IL-2 and IL-12, which promote Th1 responses for viral clearance, and IL-10, known for anti-inflammatory effects.

### PBMCs stimulation with SARS-CoV-2 peptides and spike protein

**Figure 4:**
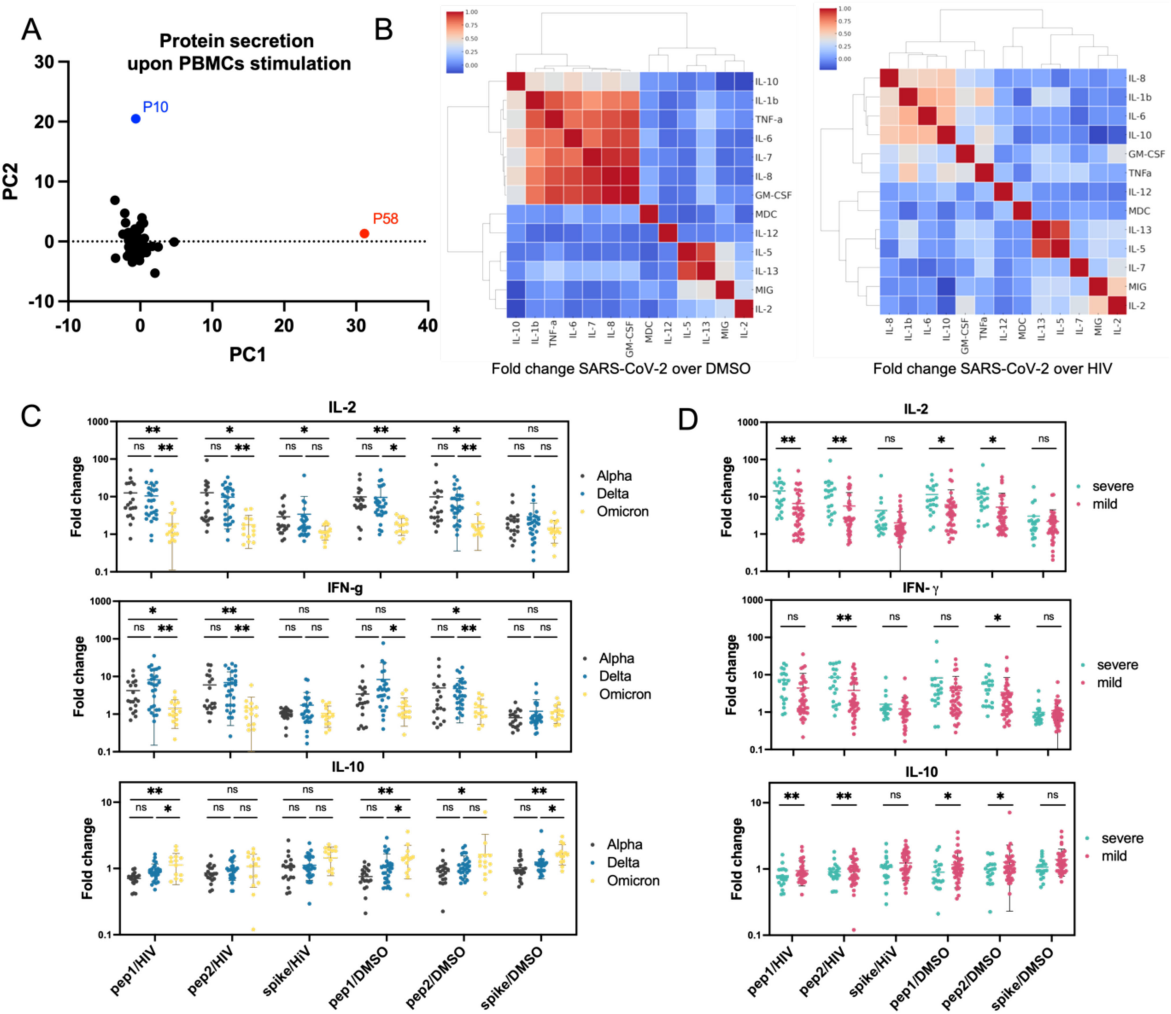
Protein secretion analysis in PBMCs from COVID-19 convalescent patients **(A)** Principal component analysis (PCA) of protein secretion profiles across all tested patients, revealing underlying patterns and variations. Most patients clustered together, with two patients appearing as outliers. **(B)** Heatmap showing hierarchical clustering of correlations between secretion levels of individual proteins following PBMC stimulation. Data are presented as the fold change in protein secretion under SARS-CoV-2 peptide stimulation compared to DMSO or HIV peptide stimulation. Red clusters highlight proteins that were not upregulated upon PBMC stimulation. **(C, D)** Secretion patterns of IL-2, IFN-γ, and IL-10 stratified by COVID-19 variant **(C)** and disease severity **(D)** upon various stimulations. The data demonstrate a progressive decrease in IL-2 and IFN-γ secretion and an increase in IL-10 secretion across different COVID-19 variants (from Alpha to Omicron). Furthermore, PBMCs from patients with severe disease exhibited higher IL-2 and IFN-γ secretion and lower IL-10 secretion compared to those with mild disease. Statistical significance is indicated for each comparison. ns (p > 0.05), * (p ≤ 0.05), or ** (p ≤ 0.01).

PBMCs collected from COVID-19 convalescent individuals at hospital discharge (day 0, t0) were stimulated with SARS-CoV-2 scrambled Spike peptides and Spike protein, then compared to PBMCs stimulated with control reagents, DMSO, or HIV peptides. The secretion of interleukins, including IFNγ and TNFα, was quantified to identify heightened reactivity in certain patient subsets. The analysis revealed significant variability in immune responses among COVID-19 convalescent individuals. Notably, the SARS-CoV-2 peptide cocktails, including scrambled Spike peptide 1 and peptide 2, generally induced higher cytokine and immunological factor levels than the full-length Spike protein, indicating stronger immune stimulation. Principal component analysis identified one significant cluster and two outliers, suggesting unique immune profiles, which may be relevant for personalized treatment strategies (**Figure 4A**). To clarify which proteins exhibited SARS-CoV-2-specific secretion (compared to HIV control) and which were part of the general viral response (compared to DMSO control), hierarchically ordered heat maps of secretion profiles were created using average values for PBMCs stimulated with SARS-CoV-2 peptide mix 1, peptide mix 2, and Spike protein (**Figure 4B**). Individual heat maps for each stimulant were also generated (**Supplementary Figure S10**). Fold-change analysis of protein concentrations relative to DMSO stimulation revealed a cluster of unchanged factors including IL-1β, IL-6, IL-7, IL-8, IL-10, TNFα, and GM-CSF (red cluster). The HIV stimulation comparison showed smaller clusters composed of pro-inflammatory cytokines IL-1β, IL-6, IL-8, and IL-10, indicating a more selective immune response. Among the highly upregulated proteins, IL-13 strongly correlated with IL-5, both linked to Th2-type immune responses and associated with allergic inflammation, asthma, and other viral lung infections like influenza and RSV (respiratory syncytial virus) (41, 42). Additionally, in PBMCs stimulated with SARS-CoV-2 peptides, MIG correlated with IL-2, highlighting their role in T-cell activation and recruitment. The clustering patterns revealed IL-6, IL-8, IL-1β, and IL-10 as a distinct group, separate from the IL-13/IL-5 and MIG/IL-2 clusters, due to their different functions. PBMC stimulation outcomes were analyzed by COVID-19 variant (**Supplementary Figure S11**), disease severity (**Supplementary Figure S12**), comorbidities (**Supplementary Figure S13**), and gender (**Supplementary Figure S14**). The analysis revealed limited correlations between protein secretion patterns and clinical features, though some significant differences were observed. IL-2 and IFN-γ secretion was comparable for Alpha and Delta variants but lower in Omicron patients, suggesting a more balanced T-cell response in Omicron, contrasting with the overactivation of interferon-related pathways seen in Alpha and Delta. Anti-inflammatory IL-10 secretion was slightly higher in Omicron cases (**Figure 4C**). When analyzing by disease severity, IL-2 and IFN-γ levels were higher in severe COVID-19 patients, indicating strong T-cell activation and a heightened immune response, while IL-10 was marginally higher in mild cases, suggesting a protective anti-inflammatory response (**Figure 4D**). These findings highlight an inverse pattern between the pro-inflammatory IL-2 and IFN-γ and anti-inflammatory IL-10, pointing to the role of inflammatory activation, particularly involving interferon-related pathways, in COVID-19 progression and severity.

### The analysis of proteases and their inhibitors in peripheral blood samples

**Figure 5:**
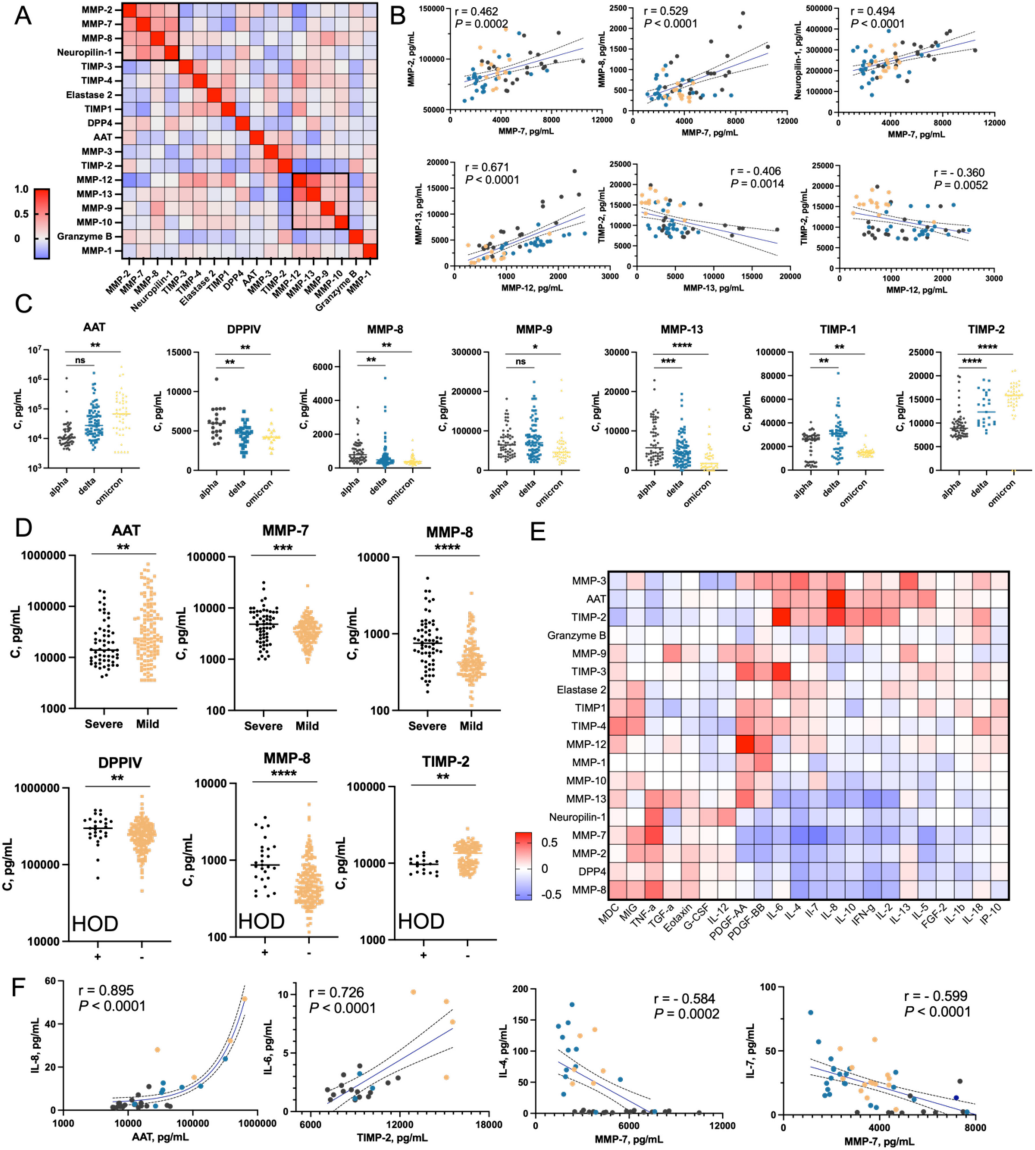
Analysis of proteases and their Inhibitors in plasma of COVID-19 convalescent patients **(A)** Correlation matrix of the abundance of selected proteases, their inhibitors, and neuropilin-1. Geometric means of protein concentrations from three time points (t0, t1, and t3) were calculated and used to determine pairwise Pearson correlation coefficients between proteins. Two clusters with the highest correlations are highlighted with black boxes (Cluster 1: MMP-2, MMP-7, MMP-8; Cluster 2: MMP-12, MMP-13, MMP-9, MMP-10). **(B)** Dot plots showing the most significant correlations between selected proteins. COVID-19 variants are color-coded: black for Alpha, blue for Delta, and yellow for Omicron. **(C)** Nested plots showing concentrations of selected proteins in individual patients, categorized by COVID-19 variant. Only proteins showing statistically significant differences between groups are presented. **(D)** Patterns of selected protein concentrations categorized by COVID-19 severity and presence of comorbidities. Only proteins with statistically significant differences between groups are shown. **(E)** Pairwise correlation matrix of concentrations of selected proteases/inhibitors and cytokines/chemokines/growth factors. The matrix was generated based on Pearson correlation coefficients calculated for each protein pair. **(F)** Dot plots presenting the most significant correlations between cytokine concentrations (y-axis) and protease/inhibitor concentrations (x-axis). For better visualization, AAT concentrations are shown on a logarithmic scale. Statistical significance is annotated as: ns (p > 0.05), * (p ≤ 0.05), ** (p ≤ 0.01), *** (p ≤ 0.001) or **** (p ≤ 0.0001).

A variety of proteases are involved in the degradation of extracellular matrix components, regulation of inflammatory responses, and tissue remodeling, all of which are critical during the recovery phase from severe viral infections like COVID-19 (11, 43). Here, we aimed to analyze the levels of a panel of proteases and their inhibitors in plasma samples from COVID-19 convalescent patients. We selected various matrix metalloproteases (MMP-1, MMP-2, MMP-3, MMP-4, MMP-7, MMP-8, MMP-9, MMP-10, MMP-12, MMP-13) and their natural inhibitors (TIMP-1 to TIMP-4). MMPs and TIMPs are critical in the remodeling and repair of tissues damaged by the virus (44). The correlation analysis between proteases and inhibitors revealed distinct clusters (**Figure 5A**). MMP-2, MMP-7, MMP-8, and neuropilin-1 were positively correlated, aligning with prior findings of elevated MMPs in severe COVID-19, linked to tissue damage and repair (**Figure 5B**) (45). MMP-12 and MMP-13 formed another cluster, negatively correlated with TIMP-2, supporting TIMPs’ role in regulating proteolytic activity to prevent tissue damage. AAT and TIMP-2 levels increased progressively across Alpha, Delta, and Omicron variants, suggesting an immune modulation response to heightened inflammation or viral pathogenicity (**Figure 5C**, **Supplementary Figure S15**). DPPIV, MMP-8, MMP-9, and MMP-13 levels decreased across COVID-19 variants, suggesting a reduced proteolytic and inflammatory response, consistent with Omicron’s lower severity compared to Alpha (46). TIMP-1 was downregulated in convalescent patients recovering from mild Omicron cases, supporting previous findings of TIMP-1’s correlation with COVID-19 severity (47). AAT levels were higher in mild cases, likely offering protection against tissue damage, while MMP-7, MMP-8, MMP-13, and neuropilin-1 were elevated in severe cases, promoting inflammation and tissue breakdown, contributing to more severe disease pathology (**Figure 5D**, **Supplementary Figure S16**). Patients with comorbidities such as hypertension, obesity, and diabetes had elevated DPPIV, MMP-8, and TIMP-4 levels, and reduced MMP-3 and TIMP-2 levels, indicating a link between these markers and the exacerbating effects of comorbidities on COVID-19 outcomes (**Figure 5D**, **Supplementary Figure S17**). The elevated markers suggest a persistent inflammatory state, while reduced MMP-3 and TIMP-2 may reflect impaired regulation. Immunosuppressive treatment increased elastase 2 and AAT levels, suggesting immune modulation (**Supplementary Figure S18**). Gender (**Supplementary Figure S19**) and lung ultrasound severity (**Supplementary Figure S20**) did not significantly affect biomarker profiles. We analyzed the correlations between proteases, their inhibitors, and cytokines/chemokines in plasma (**Figures 5E, F**). Among others, strong positive correlations were found between TIMP-2 and IL-6, and between AAT (α-1 antitrypsin) and IL-8. IL-6 and IL-8 are key inflammatory cytokines elevated in severe COVID-19, suggesting that increased TIMP-2 and AAT may protect tissues from proteolytic damage (9). Conversely, MMP-7 negatively correlated with IL-4 and IL-7, and suggesting that these proteases downregulate immune responses to prevent excessive tissue damage and immune overactivation. These findings highlight the regulatory roles of MMPs and TIMPs in balancing inflammation, immune responses, and tissue remodeling in COVID-19, providing insights into potential therapeutic strategies (48, 49).

### Identification of immunological cells subpopulations in COVID-19 convalescents by mass cytometry

**Figure 6:**
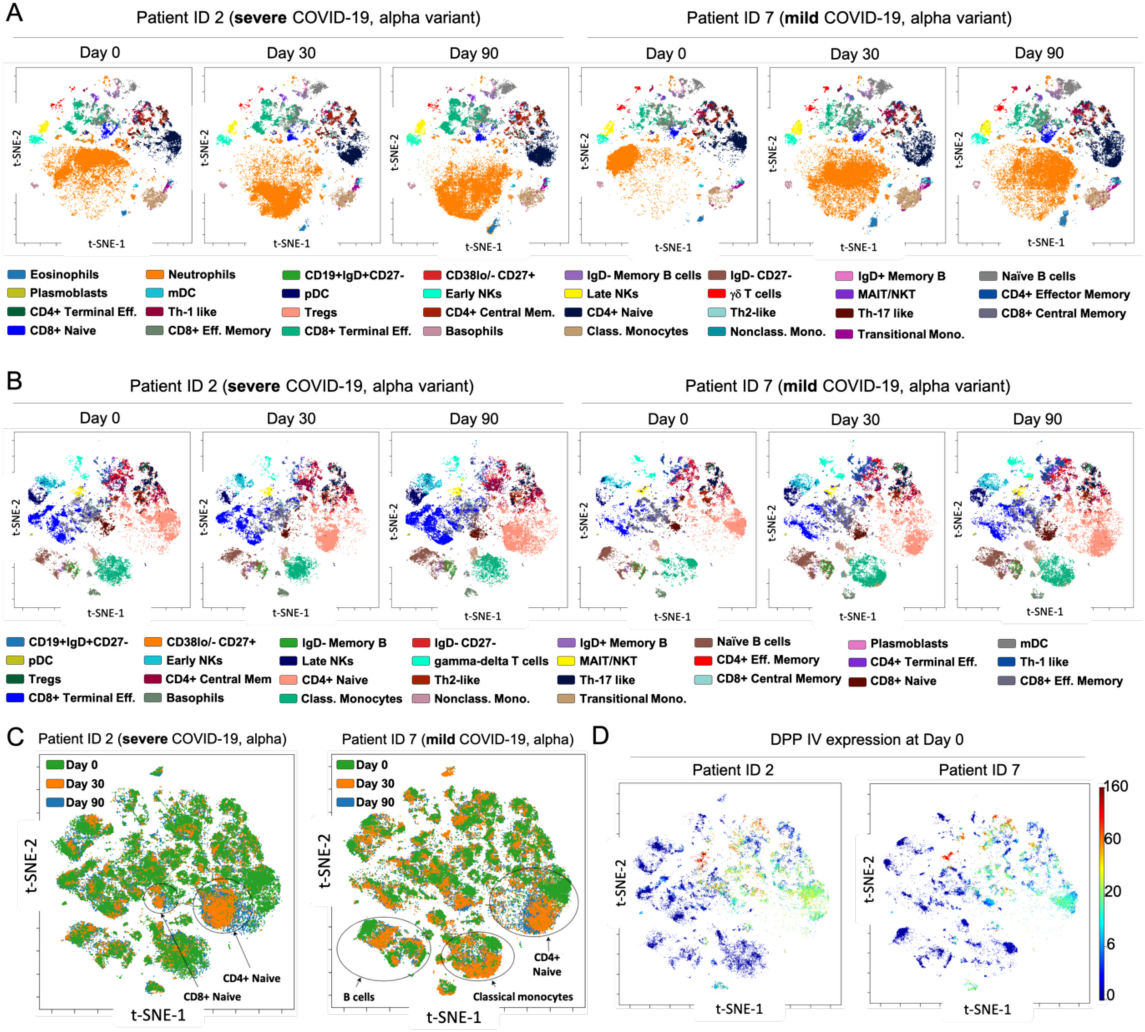
Immune profiling of COVID-19 convalescent patients by mass cytometry. **(A)** viSNE maps depicting 31 immune cell populations: granulocytes and lymphocytes in two representative patients infected with the Alpha variant (patient no. 2, severe disease; patient no. 7, mild disease) over 90-day period. **(B)** viSNE maps illustrating 29 mononuclear cell populations in representative patients with severe and mild COVID-19 alpha variant, throughout all time points. Focusing exclusively on mononuclear cells provides deeper insight into specific immune subpopulations and the distribution of markers within these compartments. For instance, CD4+ naïve and CD8+ terminal effector cells display distinct patterns of marker expression between mild and severe cases. **(C)** Aggregated viSNE maps for patients no. 2 and no. 7, showing immune cell distribution from the day of discharge (time 0) to 90 days post-recovery, within single patients. **(D)** viSNE maps demonstrating the distribution and expression levels of the DPPIV protease within mononuclear cells for patients no. 2 and no. 7 at day 0, revealed distinct patterns of expression between mild and severe cases, while involving the same immune cell populations.

Mass cytometry has become a key tool for analyzing immune responses in diseases like COVID-19 (50). Studies show that severe cases are marked by an increase in pro-inflammatory monocytes (CD169+) and neutrophils, with a decrease in CD8+ T cells, indicating immunosuppression and dysfunction. This imbalance persists during recovery, suggesting ongoing immune dysregulation. In contrast, mild cases show fewer immune disturbances and a trend towards normalization. Early, coordinated immune responses are linked to better outcomes, highlighting the critical role of timely immune activation in disease progression (50–52). We analyzed circulating immune cells in COVID-19 convalescent patients at three time points post-hospital discharge (days 0, 30, and 90). Fresh peripheral blood samples were stained and examined via mass cytometry to quantify 30 immune markers, including those related to SARS-CoV-2 (TMPRSS2 transmembrane serine protease 2, ACE2 angiotensin-converting enzyme 2, DPPIV, neuropilin-1, and furin) (53, 54). After quality control and batch normalization, we manually gated canonical immune cell populations and assessed their frequencies and SARS-CoV-2 protein expression. t-SNE dimensionality reduction revealed consistent immune cell patterns across patients, regardless of disease severity or variant, over the 90-day period (**Figure 6A**, **Supplementary Figure S21A**).

The most pronounced differences were seen at time 0 post-discharge, with neutrophils highly abundant initially but normalizing after 30 and 90 days, suggesting a gradual restoration of immune homeostasis. To further explore immune differences, we performed a second t-SNE analysis excluding neutrophils and focusing on mononuclear cells. At discharge, we observed small decreases in monocytes, CD8+ terminal effector, and CD8+ effector memory cells, but these populations increased and normalized over 90 days, indicating a return to a stable immune profile (**Figure 6B**). However, in severe Delta variant cases, mucosal-associated invariant T cells (MAIT) and natural killer T cells (NKT) showed significant and persistent reductions across all time points, suggesting these cells may be more susceptible to SARS-CoV-2-related dysregulation in more virulent strains (**Supplementary Figures S21B, C**). Comparing mild Delta and Omicron patients, immune profiles were largely similar, except for a notable decrease in γδ T cells in Omicron cases, consistent with recent studies pointing to γδ T cell depletion in Omicron. This persistent reduction might impair immune surveillance and contribute to differing disease outcomes (55), despite earlier evidence identifying their depletion as a key issue in previous variants (56). Although viSNE graphs showed similar immune signatures at days 0, 30, and 90, overlay analysis revealed subtle but significant changes in CD4+ and CD8+ naïve T cells in severe cases, as well as changes in CD4+ naïve T cells, monocytes, and B cells in mild cases (**Figure 6C**). These findings suggest that while the overall immune landscape appears stable, there are underlying cellular shifts that could impact long-term immunity and recovery outcomes. Considering SARS-CoV-2’s preference for infecting epithelial cells expressing ACE2 and TMPRSS2, we investigated whether immune cells could act as reservoirs for the virus. Our analysis revealed that among SARS-CoV-2-related proteins, only DPPIV was present at measurable levels in immune cells, primarily in MAIT/NKT cells and subsets of CD4+ and CD8+ T cells. DPPIV expression was consistent across COVID-19 severity, suggesting these immune cells play a limited role in viral propagation (**Figure 6D**). The expression of other SARS-CoV-2-related proteins was negligible, reinforcing the primary role of epithelial cells in infection (**Supplementary Figure S22**).

### Integration of mass cytometry data with cytokine and protease levels in plasma

**Figure 7:**
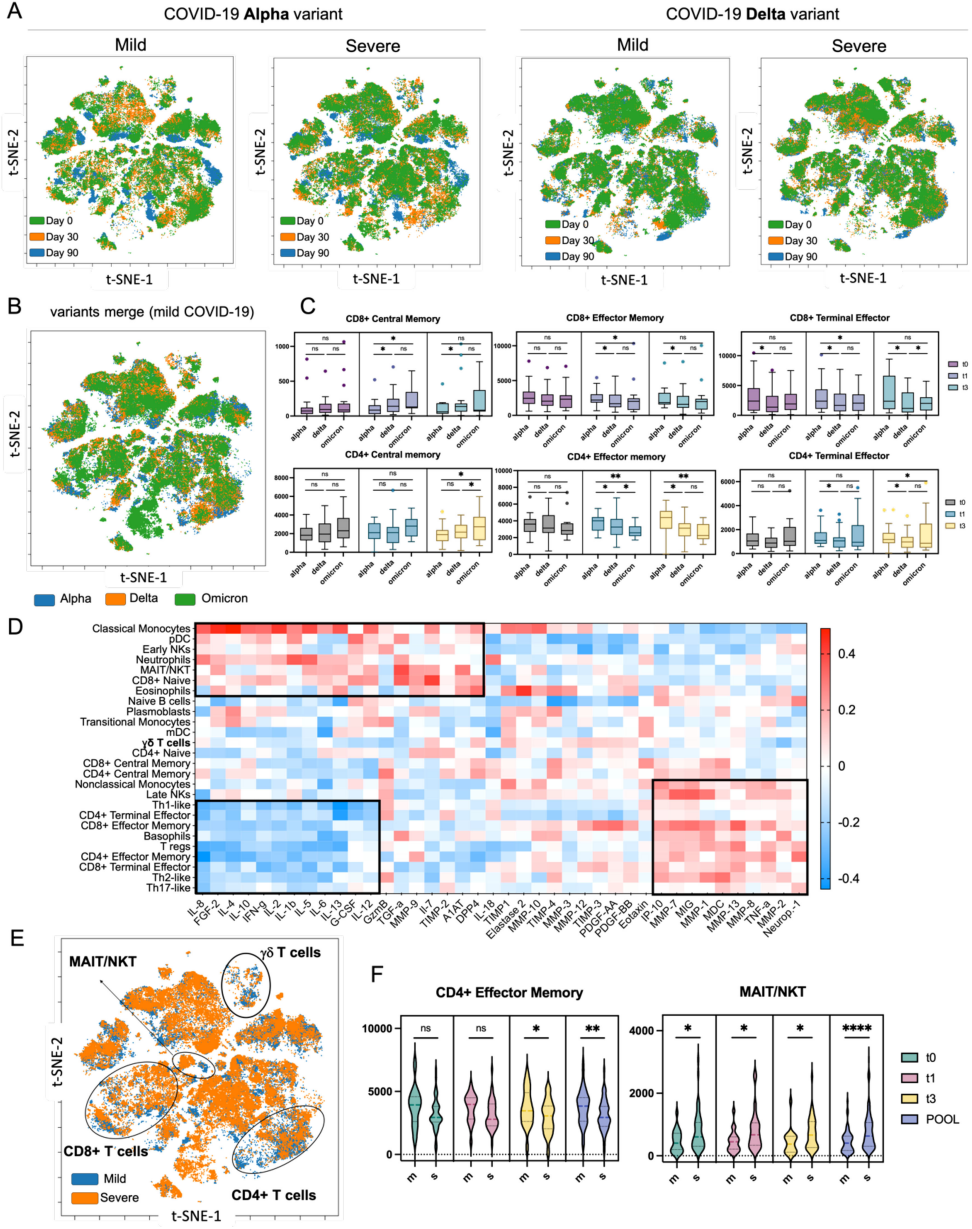
High-dimensional analysis of COVID-19 patients using mass cytometry and xMAP technologies. **(A)** Merged viSNE maps illustrating the dynamics of immune system recovery from day 0 to day 90 post-discharge, comparing Alpha and Delta variants, with aggregated data for mild and severe cases. **(B)** Aggregated viSNE map depicting immune profiles in mild COVID-19 patients across three variants: Alpha, Delta, and Omicron at t0. **(C)** Nested graphs showing the distribution of selected immune cell types that exhibited statistical significance in the analysis across different COVID-19 variants at three time points post-recovery, from day 0 to day 90. **(D)** Hierarchically ordered correlation matrix displaying the relationships between immune cell populations and concentrations of cytokines, chemokines, proteases, and their inhibitors in plasma samples at t0. The heatmap revealed three distinctive clusters. The first and third clusters showed correlations between immune cells and MMPs, suggesting their potential role in tissue repair. **(E)** Aggregated viSNE map highlighting differences in immune cell composition during the recovery process in patients with mild versus severe COVID-19 at t0. **(F)** Nested graphs illustrating the counts of CD4+ effector memory T cells and MAIT/NKT, which exhibited significant changes as revealed by the t-SNE analysis, cells based on COVID-19 severity, across all investigated time points. ns (p > 0.05), * (p ≤ 0.05), *** (p ≤ 0.001) or **** (p ≤ 0.0001).

Our mass cytometry analysis across different COVID-19 variants and severities revealed distinct differences that could influence recovery trajectories. By merging and overlaying viSNE maps based on severity and variant at three time points, we found that Alpha variant convalescent patients exhibited more pronounced changes in monocytes, CD4+, and CD8+ T cells during recovery, whereas Delta variant patients showed a more stable immune signature over 90 days (**Figure 7A**). Cell enumeration confirmed a progressive decrease in CD8+ and CD4+ effector memory cells from Alpha to Omicron variants, alongside an increase in central memory cells (**Figures 7B, C**). Omicron patients also had lower myeloid dendritic cells (mDCs) and higher plasmacytoid dendritic cells (pDCs) compared to Alpha patients (**Supplementary Figure S23**). These differences were most significant at time 0, with marked normalization by day 30, indicating a robust immune recovery within this period. Importantly, Omicron patients were classified only as mild cases, and therefore no comparative analysis (mild vs severe) was possible.

To gain a deeper mechanistic understanding of COVID-19 recovery, we combined immune signature data from mass cytometry with cytokine levels and protease/inhibitor data through a correlative and hierarchical analysis (**Figure 7D**). Both immune cell populations and cytokine and protease/inhibitor profiles were selected based on their significant differences between mild and severe cases. This revealed three major clusters: the first showed a positive correlation between monocytes, dendritic cells, NK cells, MAIT/NKT cells, and neutrophils with pro-inflammatory cytokines (e.g., IFNγ, IL-1β, IL-6), indicating a strong inflammatory response. The second cluster revealed a negative correlation between subsets of CD4+ and CD8+ T cells and these cytokines, suggesting a regulatory mechanism to control inflammation. The third cluster indicated a positive correlation between CD4+ and CD8+ T cells with MMPs and chemokines, pointing to their role in tissue repair during recovery. Aggregated viSNE maps distinctly separated immune signatures from mild and severe cases, revealing differences in CD4+, CD8+, MAIT/NKT and γδ T cells (**Figure 7E**). The detailed analysis indeed revealed that the number of CD4+ effector memory T cells are higher in patients with mild COVID-19, whereas the number of MAIT/NKT increased with COVID-19 severity (**Figure 7F**, **Supplementary Figure S24**). In summary, our comprehensive analysis using mass cytometry revealed distinct immune signatures in COVID-19 convalescent patients, which varied by disease severity and viral variant.

### The longitudinal analysis of convalescent patients after two years post recovery

**Figure 8:**
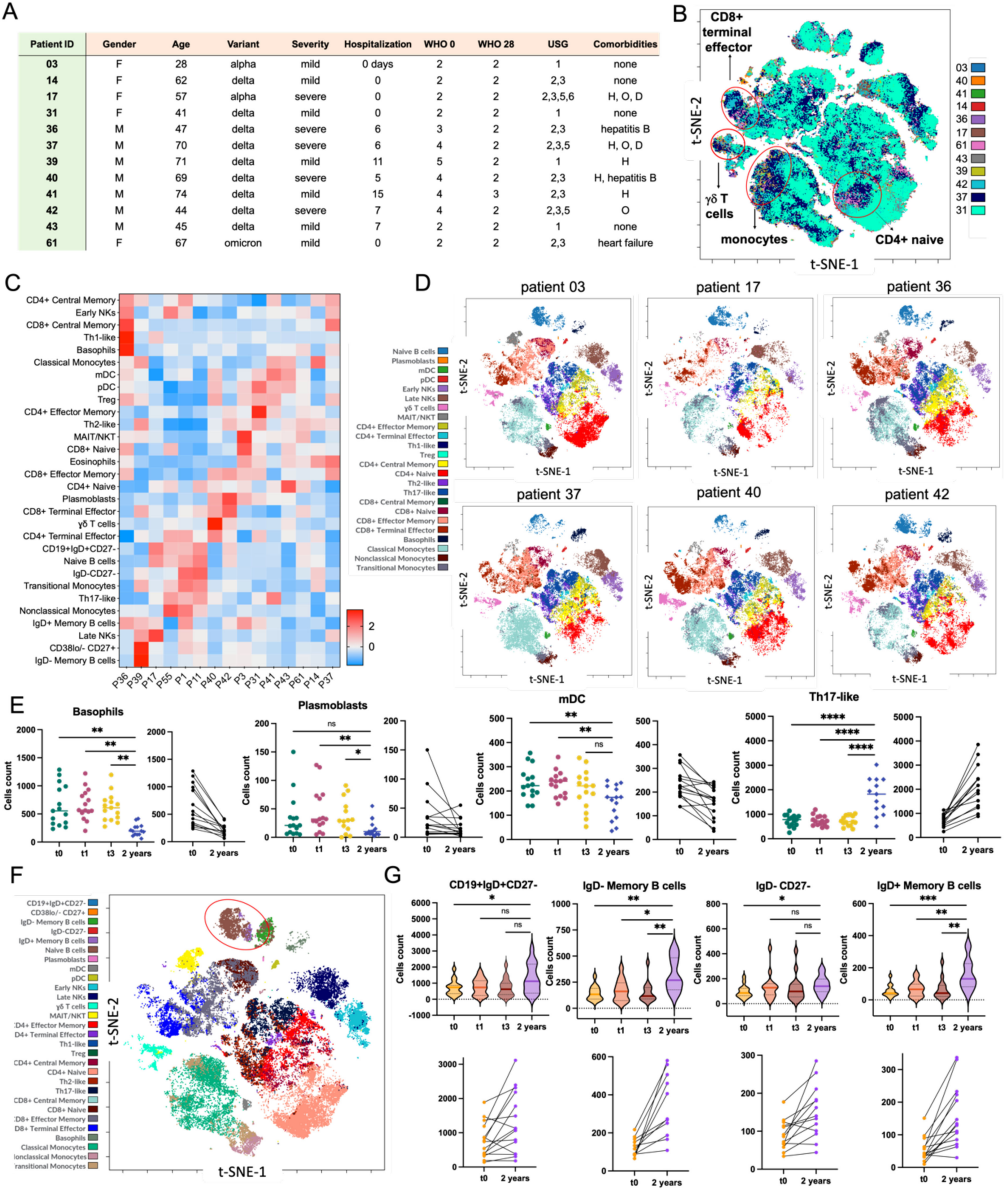
Mass cytometry analysis of the immune system trajectory in COVID-19 convalescent patients 2 years post-recovery. **(A)** Summary table of the clinical characteristics of patients included in the 2-year follow-up study (H – hypertension, O – obesity, D – diabetes). **(B)** Aggregated viSNE map of mononuclear cells from 12 patients, showcasing the diverse composition of γδ T cells, classical and transitional monocytes, and various subpopulations of CD4+ and CD8+ cells. **(C)** The heatmap presented has been Z-scored and hierarchically clustered to identify patterns of immune signatures across patients (Px) **(D)** Individual viSNE maps of mononuclear cells (lymphocytes, monocytes, and dendritic cells) for selected patients 2 years post-recovery. Patient 03, who had mild COVID-19, was grouped with patients who had severe COVID-19 based on lung ultrasound examination and hospitalization status (no 17, 36, 37, 40, and 42) with the following distribution of variants: Alpha in 17 cases, and Delta in the remaining samples. **(E)** Nested graph showing changes in the counts of selected cell types from day 0 (discharge from the hospital) to the 2-year follow-up. Scatter plots illustrate the trends in cell count changes for individual patients over two years. **(F)** viSNE map for individual patients, depicting the landscape of mononuclear cells 2 years post-recovery, with a particular focus on B cells, including memory cells (red circle). **(G)** Nested violin plot and scatter plots demonstrating the increasing counts of selected memory cells (CD19+IgD+CD27-; IgD- memory B cells; IgD-CD27-; IgD+ memory B cells) over the 2-year period following hospital discharge. Statistical significance is annotated as: ns (p > 0.05), * (p ≤ 0.05), ** (p ≤ 0.01), *** (p ≤ 0.001) or **** (p ≤ 0.0001).

High-dimensional, single-cell analysis of COVID-19 convalescent patients has provided insights into immune recovery dynamics, but the long-term effects on the immune system remain unclear. Recent studies show persistent immune changes, such as dysregulation in T-cells, B-cells, and monocytes up to 10 months post-infection (57), and altered immune cell composition for up to 6 months (58). These findings highlight the need for longitudinal studies. To further investigate long COVID, we performed mass cytometry on 15 patients from our cohort, 2 years post-recovery. Most (9 out of 15) were infected with the Delta variant, with a range of disease severity and diverse medical backgrounds (**Figure 8A**).

We first analyzed peripheral blood by mass cytometry, identifying over 30 immune cell populations. The viSNE maps showed a generally similar immune landscape across the group, but notable changes in CD4+ naive T cells, CD8+ terminal effector T cells, γδ T cells, and monocytes (**Figure 8B**). These findings suggest that, even two years post-infection, certain immune cell populations may still show persistent alterations, possibly indicating ongoing immune dysregulation or slow recovery. In particular, changes in γδ T cells and monocytes may reflect a lingering inflammatory response or altered immune readiness, as observed in previous studies (59). Given the slight variations in viSNE maps, we conducted a more detailed clustering analysis to identify patterns in immune cell populations (**Figure 8C**), revealing long-term alterations in specific subsets. We then compared the viSNE map of Patient 03, a 28-year-old female with mild COVID-19 Alpha variant (no hospitalization, normal lung ultrasound, no comorbidities), to those of patients with severe COVID-19, hospitalization, and lung changes (**Figure 8D**). Multidimensional analysis showed that Patient 03 had twice the CD8+ naive T cells and five times the MAIT/NKT cells compared to the average, suggesting that a higher presence of these cells might be linked to quicker recovery and a more efficient immune response (57).

Next, we examined over 30 immune cell subtypes at four time points (t0, t1, t3, and 2 years post-recovery). The analysis revealed that basophils, plasmablasts, mDC cells, and early NK cells decreased over time, reaching their lowest levels after two years (**Figure 8E**, **Supplementary Figure S25**). Other cells, like classical monocytes and Tregs, also showed a tendency to decrease, while some cell types remained unchanged. These findings align with previous studies, indicating that these declines likely reflect a gradual normalization of the immune system. This is further supported by the increase in memory B cells after two years, suggesting a return to immune stability (60). Finally, recognizing the importance of memory B cells in long-term immunity against SARS-CoV-2, we focused on these cell types. Memory B cells, including CD19+IgD+CD27- naive B cells, IgD- memory B cells, and IgD+ memory B cells, play a key role in preventing severe reinfectionv (36, 59). Our analysis (**Figure 8F**) showed that these memory B cell populations increased two years post-recovery compared to earlier time points (t0 to t3), while IgD-CD27- cells remained stable (**Figure 8G**). This suggests that the immune system continues to adapt after recovery, potentially enhancing protection against future SARS-CoV-2 exposures (58).

## Discussion

Our study provides significant insights into the prolonged immune and inflammatory responses in individuals recovering from COVID-19, particularly in relation to protease activity and immune cell dynamics. The longitudinal nature of our research, extending up to two years post-infection, allows for a deeper understanding of how these processes evolve over time and influence long COVID outcomes. Our study highlights the impact of comorbidities on immune recovery in COVID-19 patients. Patients with underlying conditions, such as hypertension, obesity, and diabetes, demonstrated distinct biomarker profiles, with higher levels of inflammatory markers and proteases. This underscores the need for tailored therapeutic interventions that address both the viral infection and the broader metabolic and inflammatory challenges faced by these patients​

One of the key findings of this study is the persistent immune dysregulation observed in severe cases of COVID-19, particularly in individuals infected with the Delta variant. This is consistent with other studies that have shown that severe COVID-19 often leads to long-term immune disturbances, including in T cell and monocyte populations. Our analysis further emphasizes that specific proteases, such as elastase 2 and granzyme B, as well as their inhibitors like alpha-1 antitrypsin (AAT), play crucial roles in the recovery process. The balance between these enzymes and their inhibitors, particularly elastase 2 and AAT, appears to be important for managing inflammation and promoting effective tissue repair. Dysregulation of this balance could lead to either inadequate healing or excessive fibrosis, contributing to the persistence of long COVID symptoms in some patients. Our findings concerning DPPIV and granzyme B underscore their roles in modulating the immune response during recovery. Granzyme B, known for its role in inducing apoptosis in infected cells, helps control viral spread, while DPPIV, through its dual function in immune modulation and glucose metabolism, might have long-term implications for metabolic health in post-COVID-19 recovery​. These insights extend beyond previous studies by connecting protease activity not only to immediate immune responses but also to the prolonged inflammatory profiles observed in long COVID-19 patients. Moreover, our results point to the dynamic regulation of matrix metalloproteinases (MMPs) and their tissue inhibitors (TIMPs). Elevated levels of MMP-7, MMP-9, and MMP-13 in severe COVID-19 cases are indicative of ongoing tissue remodeling and inflammation, which could contribute to lung fibrosis and other long-term complications. The correlation between MMPs and inflammatory cytokines, such as IL-6 and IL-8, further supports the hypothesis that protease activity is intimately linked to the immune response, particularly in the context of severe tissue damage​

Another important aspect of our research involves the mass cytometry analysis, which provided a comprehensive view of immune signatures across varying severities of disease and viral variants. The gradual normalization of immune cell populations observed in patients with mild COVID-19, in contrast to the persistent alterations seen in severe cases, highlights the importance of early and coordinated immune responses for better recovery outcomes. Specifically, the reduction in CD8+ effector memory T cells and altered expression of DPPIV in MAIT/NKT cells aligns with the broader literature on immune exhaustion and inflammatory dysregulation in severe COVID-19 Interestingly, our data suggest that recovery trajectories are not uniform across variants, with Delta variant convalescent patients showing a more stable immune response compared to Alpha variant patients. This may indicate a need for variant-specific therapeutic approaches to better support immune recovery in long COVID cases.

In summary, the prolonged dysregulation of protease activity and immune responses observed in our cohort contributes to the growing body of evidence that long COVID is characterized by a complex interplay between immune exhaustion, inflammatory signaling, and tissue repair mechanisms. Future research should focus on exploring therapeutic strategies that can restore this balance, potentially through targeted inhibition of protease activity or modulation of key immune pathways. These findings offer new avenues for addressing the long-term health impacts of COVID-19, particularly in high-risk populations with pre-existing comorbidities.

### Limitation of the study

While our study provides valuable insights into the long-term immune responses of COVID-19 convalescent patients, several limitations should be acknowledged. First, the sample size, particularly in the two-year follow-up cohort, is relatively small, which may limit the generalizability of our findings. Additionally, the study cohort predominantly comprises patients from a single geographic region, potentially introducing biases related to local healthcare practices, virus variants, and demographic factors. Another limitation is the lack of a control group of uninfected individuals, which would have provided a clearer baseline for comparison and a better understanding of the specific immune alterations attributable to SARS-CoV-2. Despite these limitations, our study contributes significantly to the understanding of post-COVID-19 recovery, particularly with its rare two-year follow-up period.

## Data Availability

The datasets presented in this study can be found in online repositories. The names of the repository/repositories and accession number(s) can be found in the article/supplementary material.
