# Supplementary material for: Understanding the long-term interplay of SARS-CoV-2 immune and inflammatory responses with proteases in COVID-19 recovery: a longitudinal study
Source: Front Immunol. 2025 Jun 10;16:1517933. doi: 10.3389/fimmu.2025.1517933 (PMC12185401; doi:10.3389/fimmu.2025.1517933)
Supplement: Supplementary file 1 [file DataSheet1.pdf]

Understanding long-term SARS-CoV-2 immune and inflammatory responses and proteases interplay in individuals recovering from COVID-19: a longitudinal study

Natalia Ćwilichowska-Puślecka[1], Aleksandra Makowiecka[1], Małgorzata Kalinka[1], Katarzyna Groborz[1], Tobiasz Puślecki[2], Marcin Drąg[1], Krzysztof Simon[3,4], Krystyna Dąbrowska[5], Monika Pazgan-Simon[4], Marcin Poręba[1,5]

[1]Faculty of Chemistry, Wroclaw University of Science and Technology, 50-370 Wroclaw, Poland; [2]Department of Systems and Computer Networks, Wroclaw University of Science and Technology, 50-370 Wroclaw, Poland; [3]Department of Infectious Disease and Hepatology, Wroclaw Medical University, Wroclaw, Poland; [4]Department of Infectious Diseases, Regional Specialist Hospital, Wroclaw, Poland; [5]Faculty of Medicine, Wroclaw University of Science and Technology, 50-370 Wroclaw, Poland,

email: natalia.cwilichowska@pwr.edu.pl, monikapazgansimon@gmail.com, marcin.poreba@pwr.edu.pl

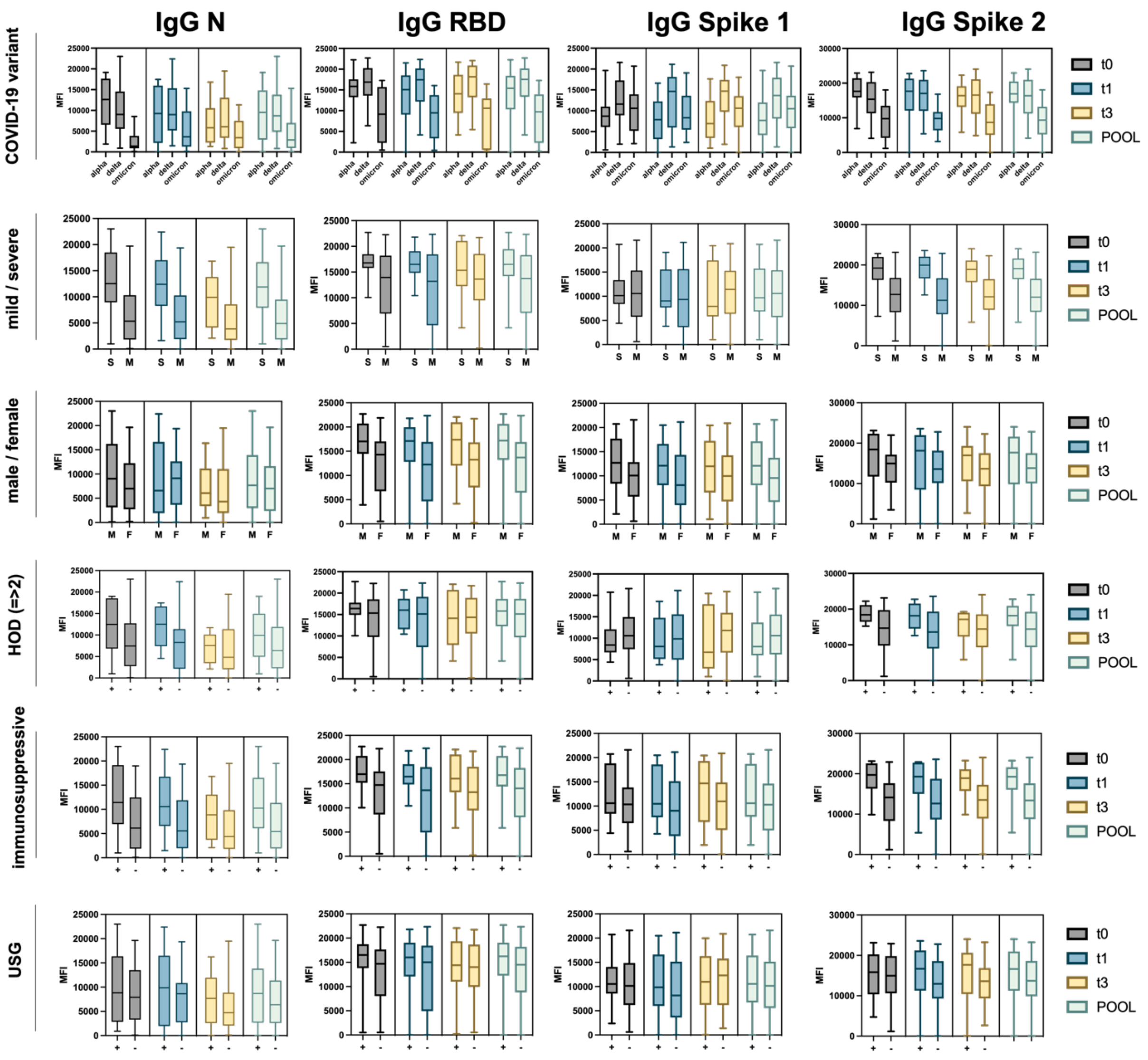

Figure S1 Box plots presenting temporal patterns of anti-SARS-CoV-2 IgG N, IgG RBD, IgG Spike1, and IgG Spike2 antibodies, categorized by COVID-19 variant, severity, gender, comorbidities, immunosuppressive treatment, and lung ultrasound (USG) examination. The y-axis presents the MFI value representing the level of antibodies.

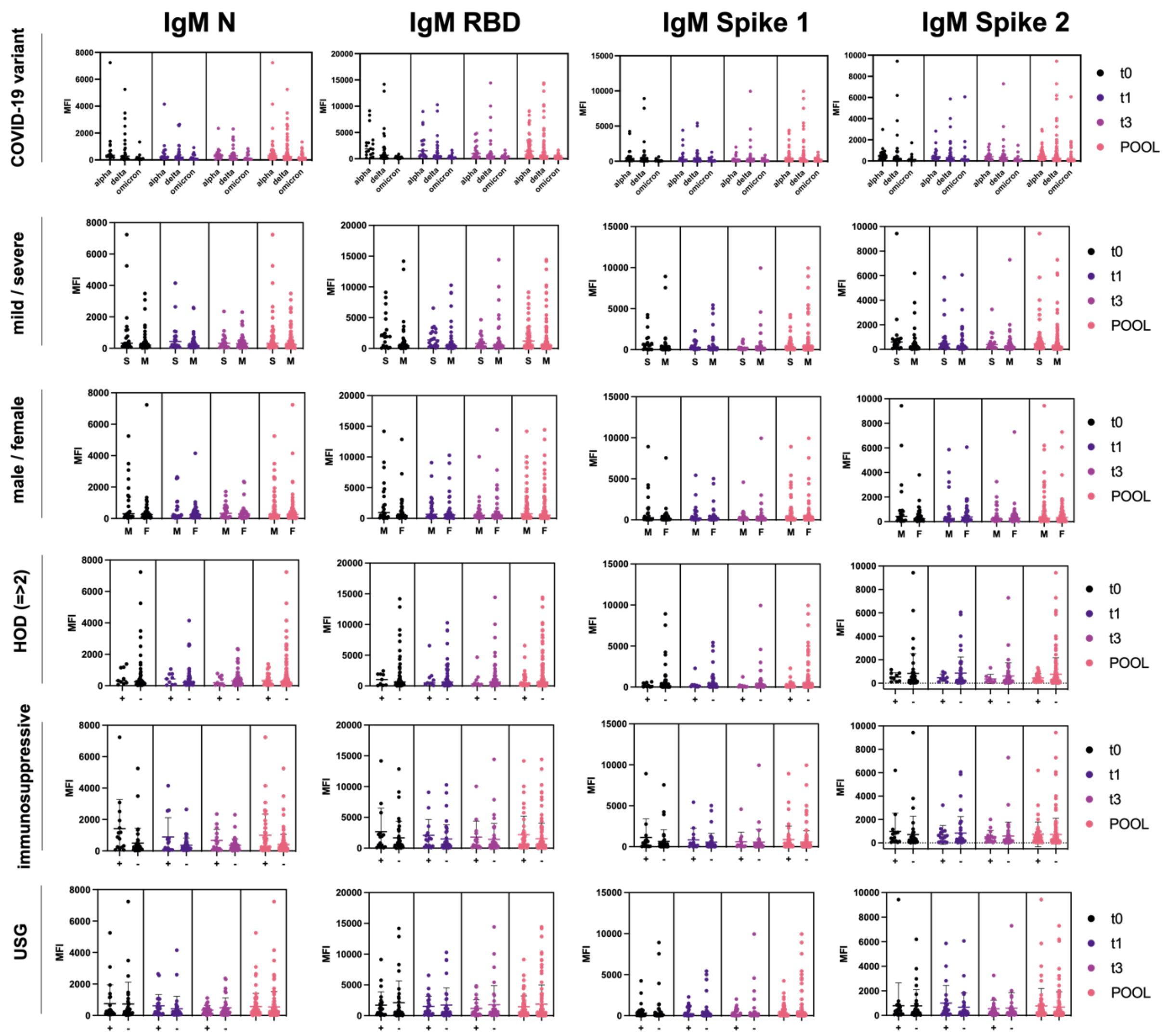

Figure S2 Dot plots presenting temporal patterns of anti-SARS-CoV-2 IgM N, IgM RBD, IgM Spike1, and IgM Spike2 antibodies, categorized by COVID-19 variant, severity, gender, comorbidities, immunosuppressive treatment, and lung ultrasound examination (USG). The y-axis presents the MFI value representing the level of antibodies.

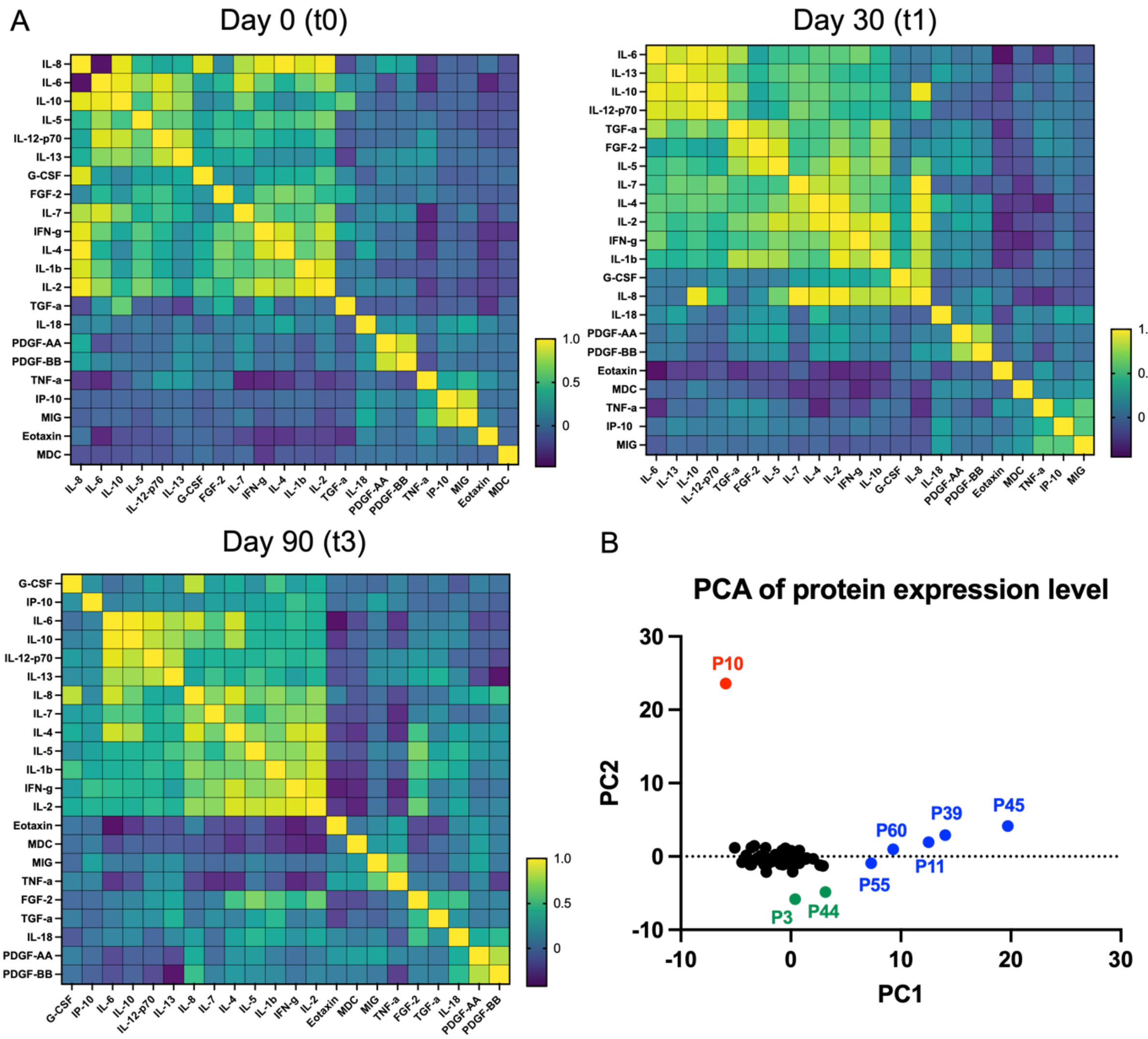

Figure S3 (A) Hierarchically ordered correlation matrix presented as Z-scored heatmaps for the aggregated analysis of cytokines, chemokines, and growth factors in COVID-19 convalescent individuals at three time points (day 0, day 30, and day 90). (B) PCA analysis of COVID-19 convalescent patients based on cytokine, chemokine, and growth factor levels, identifying patients who deviate from the main group (outliers).

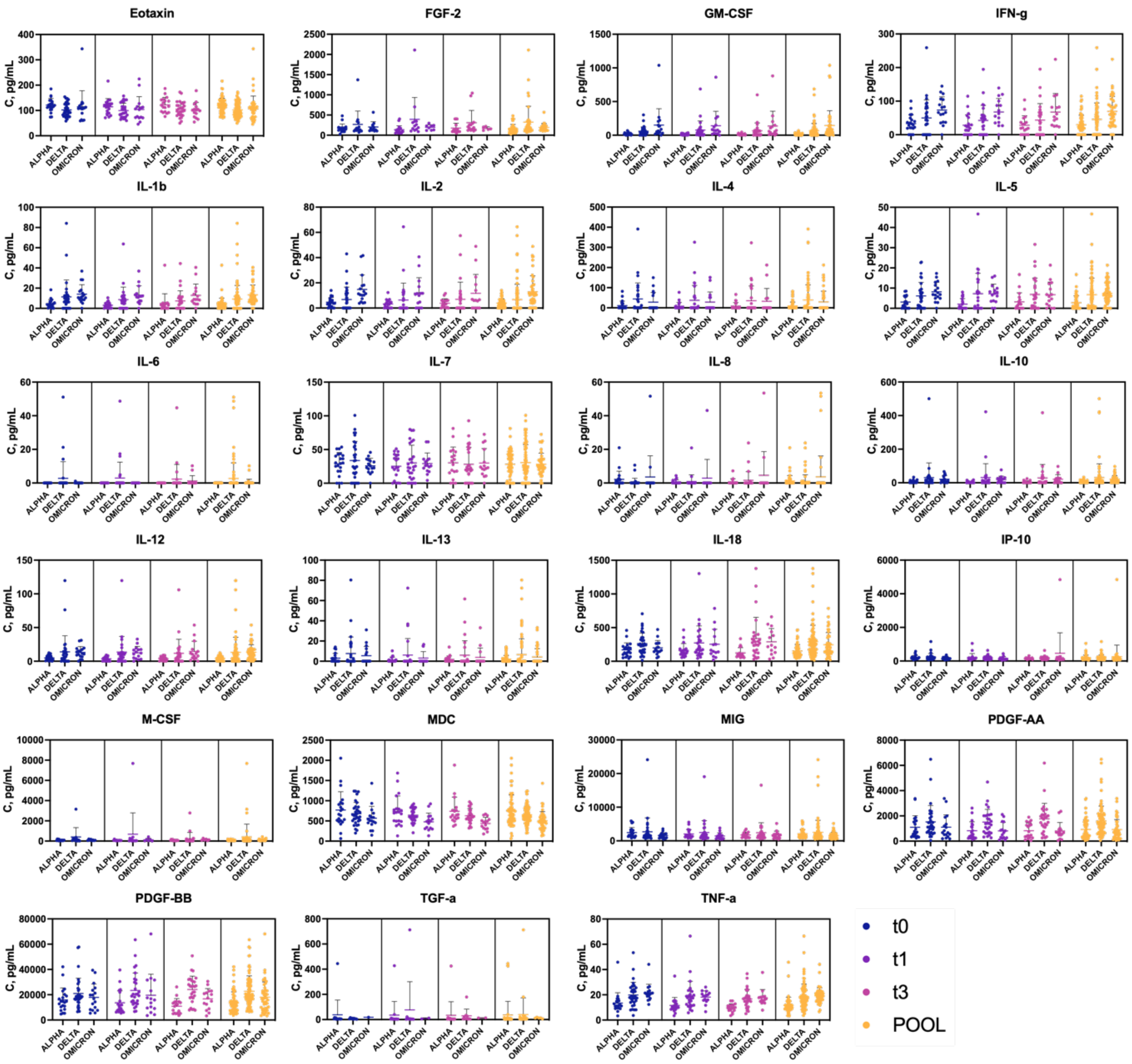

Figure S4 Nested dot graphs presenting the levels of cytokines, chemokines, and growth factors (in pg/mL) at three time points (t0, t1, t3) in COVID-19 convalescent individuals, categorized by COVID-19 variant.

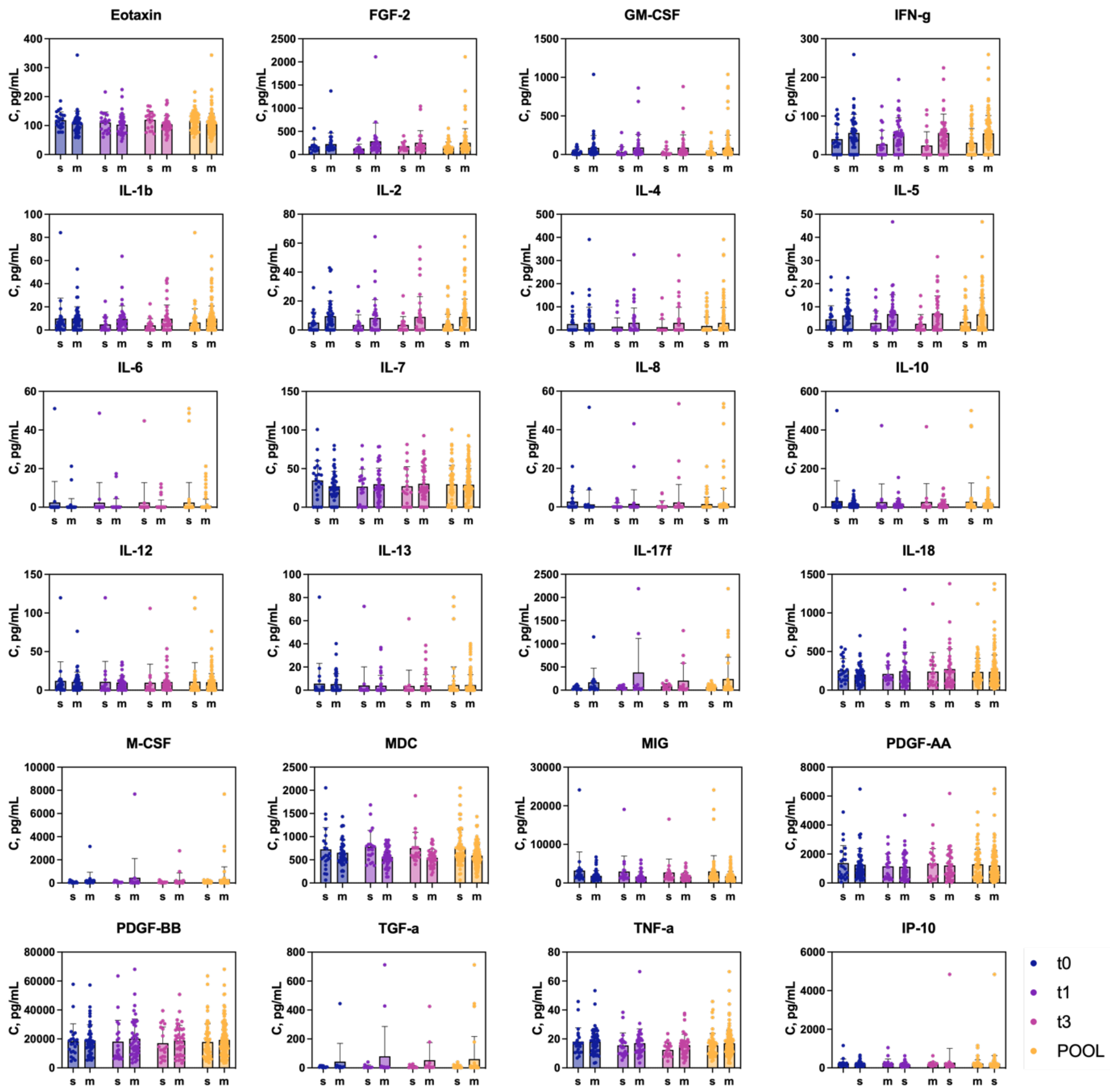

Figure S5 Nested bar graphs presenting the levels of cytokines, chemokines, and growth factors (in pg/mL) at three time points (t0, t1, t3) in COVID-19 convalescent individuals, categorized by COVID-19 severity (m – mild, s – severe).

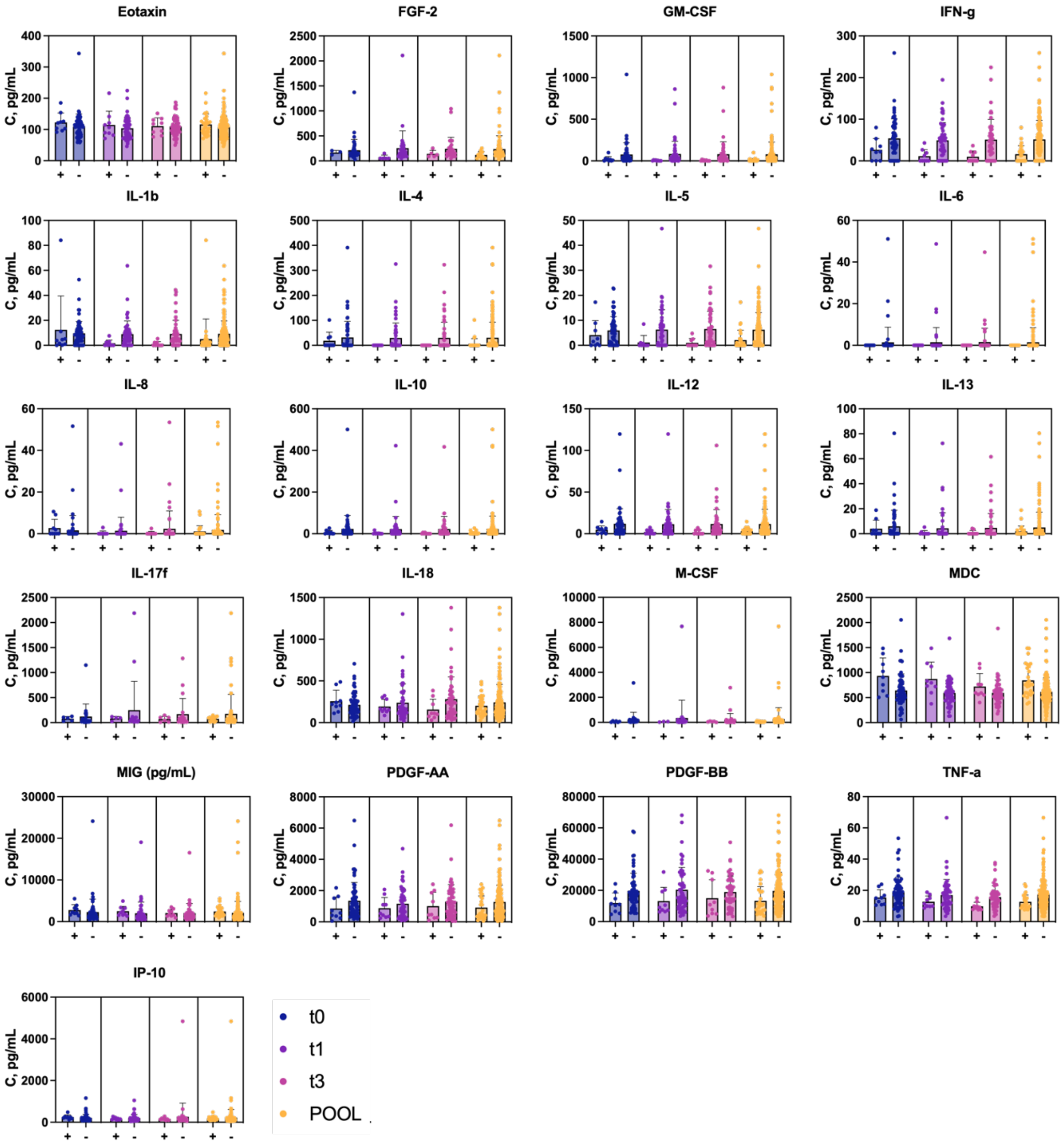

Figure S6 Nested bar graphs presenting the levels of cytokines, chemokines, and growth factors (in pg/mL) at three time points (t0, t1, t3) in COVID-19 convalescent individuals, categorized by the number of comorbidities (0-1 -, 2-3 +; obesity, hypertension, diabetes).

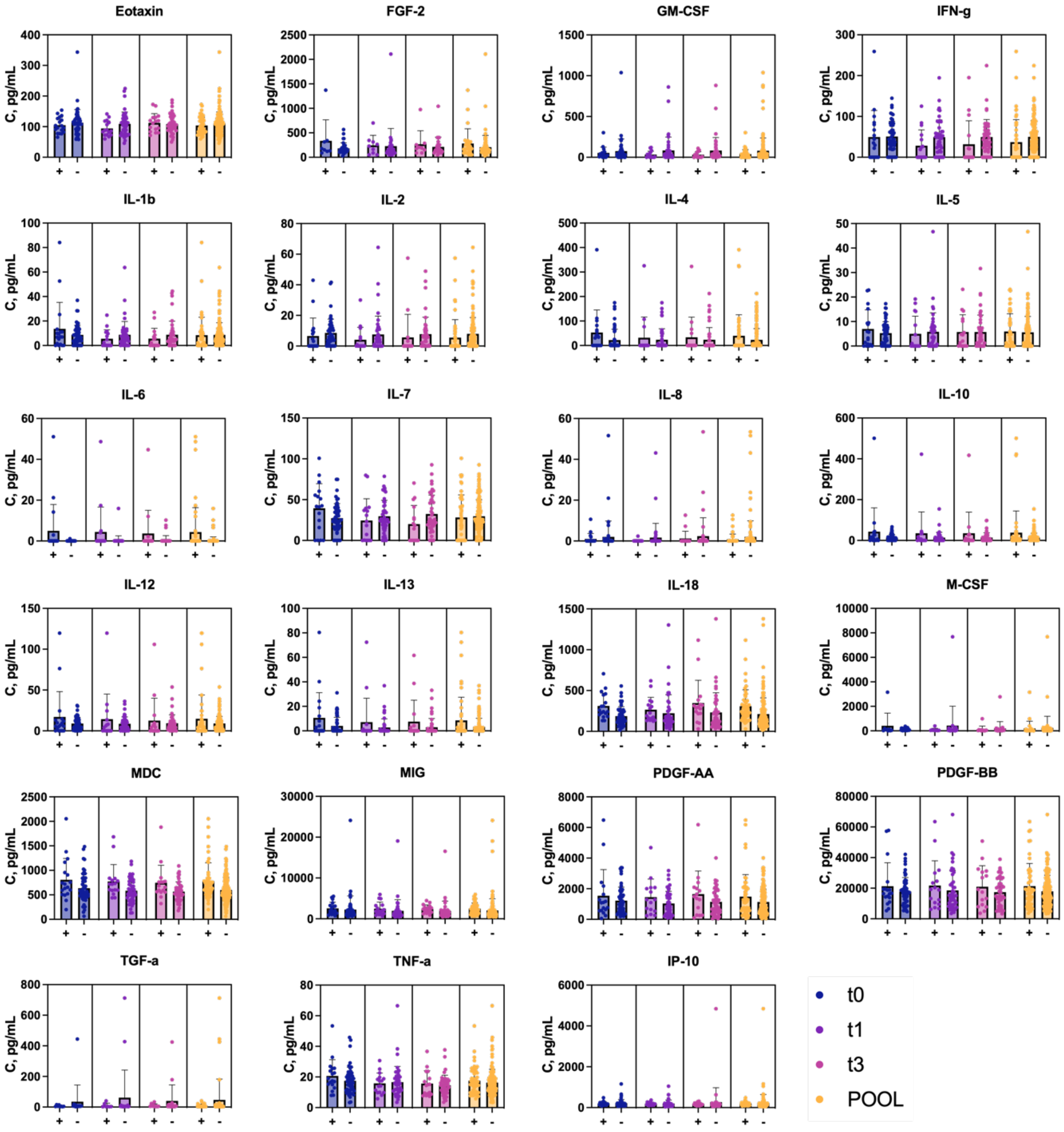

Figure S7 Nested bar graphs presenting the levels of cytokines, chemokines, and growth factors (in pg/mL) at three time points (t0, t1, t3) in COVID-19 convalescent individuals, categorized by immunosuppressive treatment (+/-).

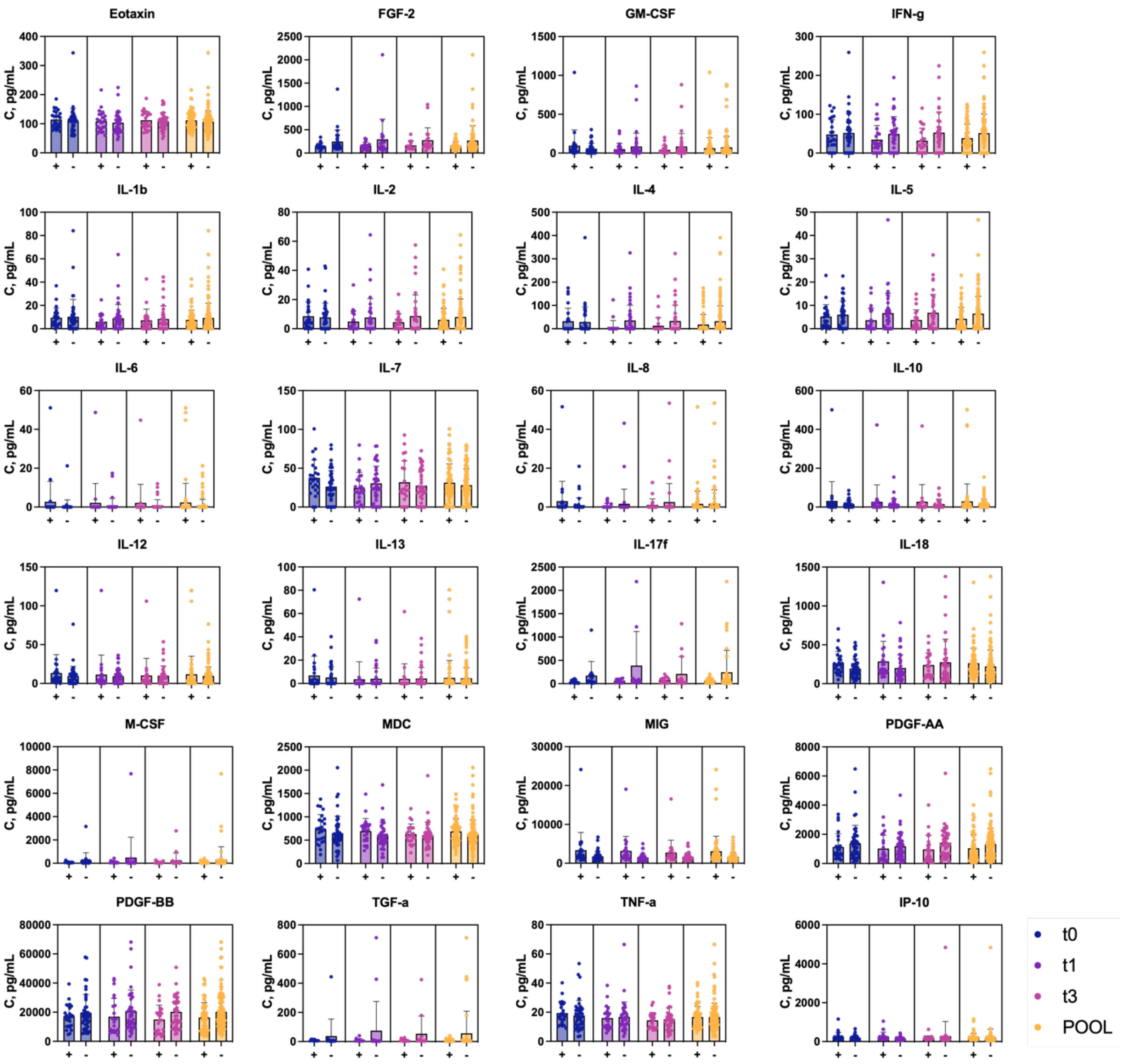

Figure S8 Nested bar graphs presenting the levels of cytokines, chemokines, and growth factors (in pg/mL) at three time points (t0, t1, t3) in COVID-19 convalescent individuals, categorized by lung ultrasound results (- no changes, + negative changes).

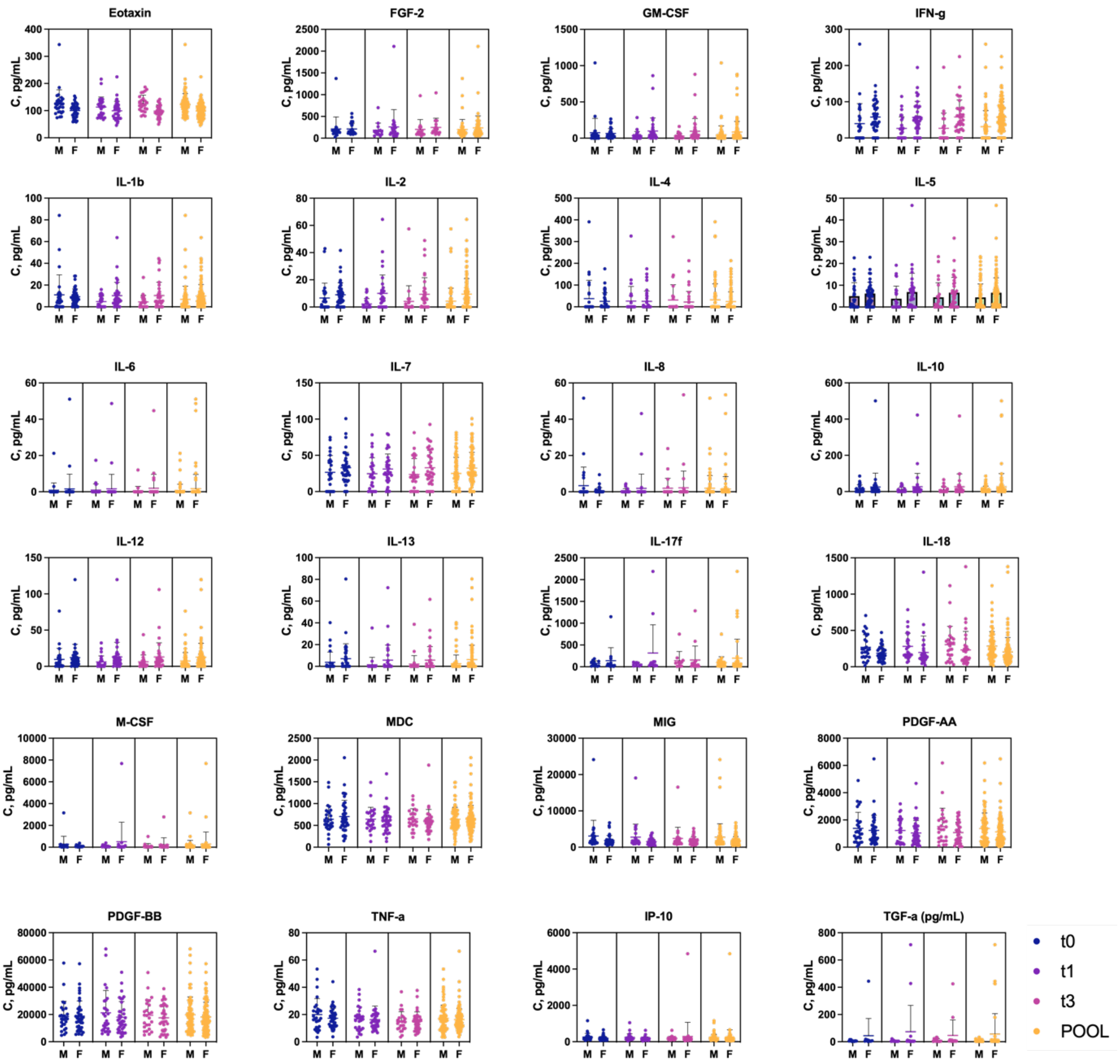

Figure S9 Nested dot graphs presenting the levels of cytokines, chemokines, and growth factors (in pg/mL) at three time points (t0, t1, t3) in COVID-19 convalescent individuals, categorized by gender (M – male, F – female).

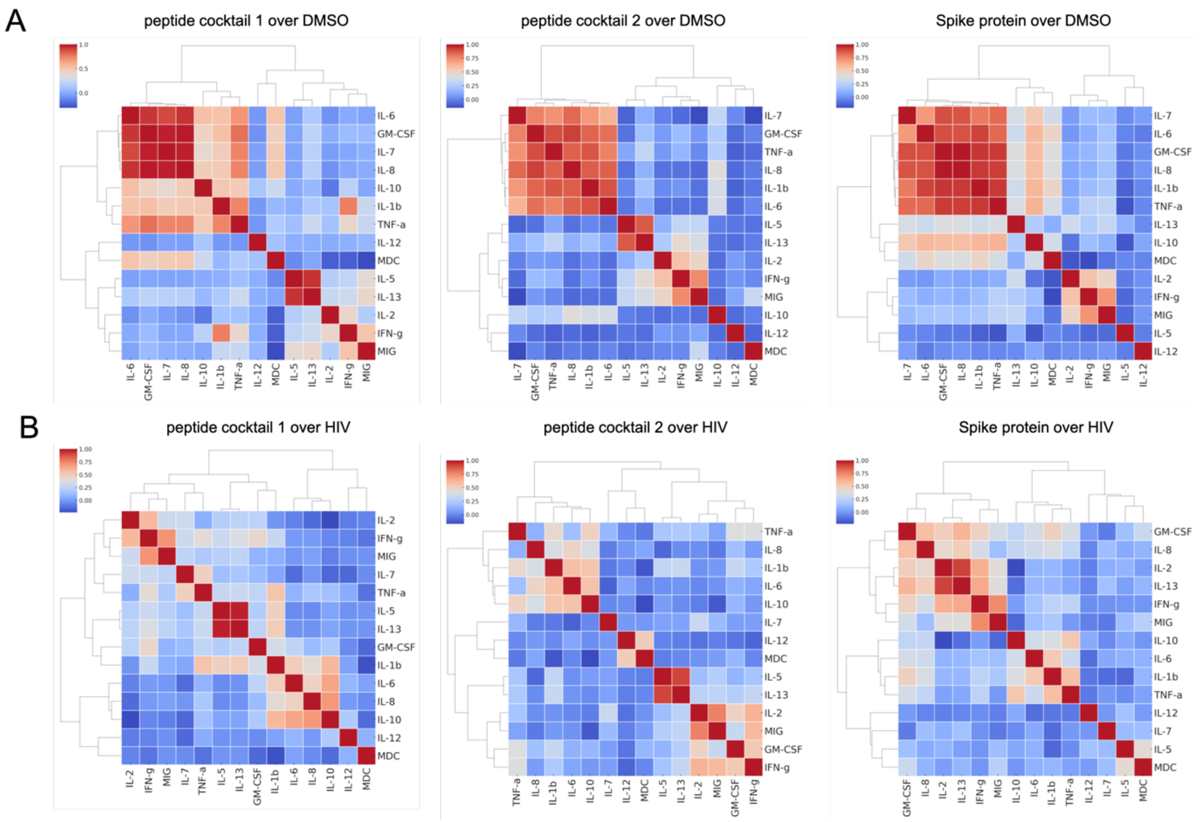

Figure S10 Hierarchically ordered correlation matrices presented as Z-scores for the analysis of protein secretion upon stimulation of PBMCs with SARS-CoV-2-derived peptides and Spike protein. (A) Matrices present the change in protein secretion from PBMCs relative to DMSO stimulation. (B) Matrices present the change in protein secretion from PBMCs stimulated with SARS-CoV-2 material relative to HIV peptide stimulation.

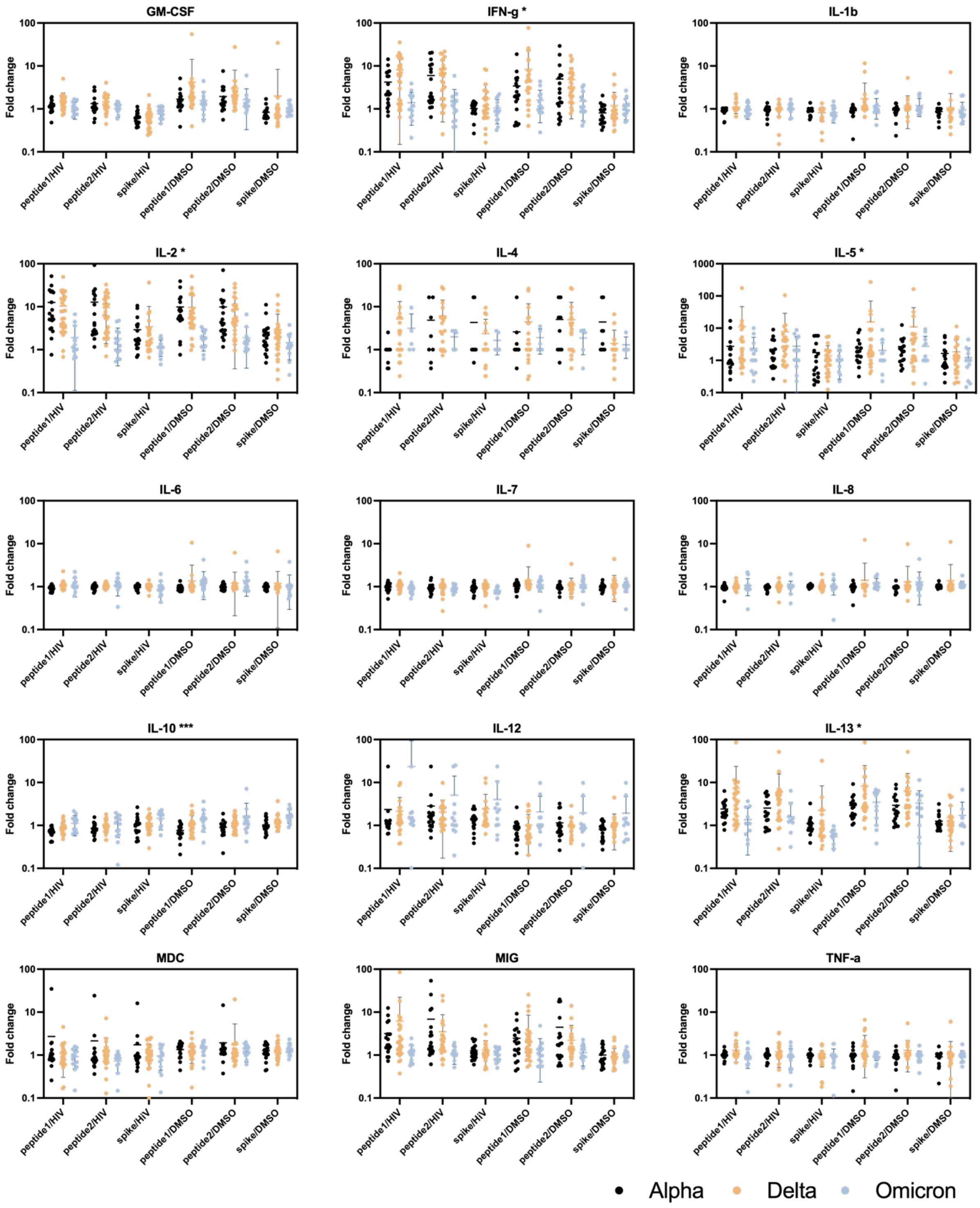

Figure S11 Dot graphs presenting protein secretion (as fold change) upon stimulation of PBMCs with SARS-CoV-2-derived peptides and Spike protein, relative to protein secretion upon DMSO or HIV peptide treatment, categorized by COVID-19 variant.

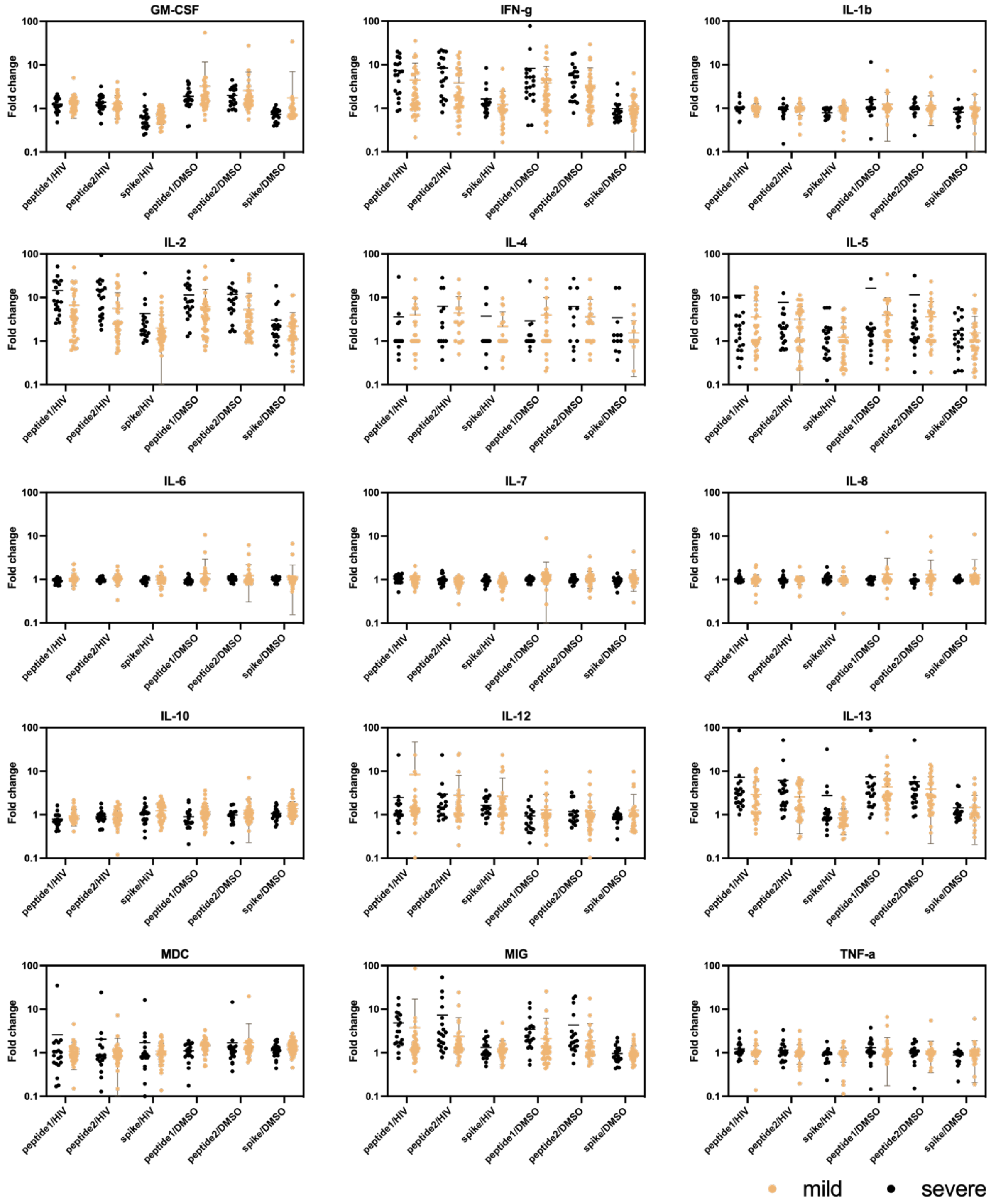

Figure S12 Dot graphs presenting protein secretion (as fold change) upon stimulation of PBMCs with SARS-CoV-2-derived peptides and Spike protein, relative to protein secretion upon DMSO or HIV peptide treatment, categorized by COVID-19 severity (mild/severe).

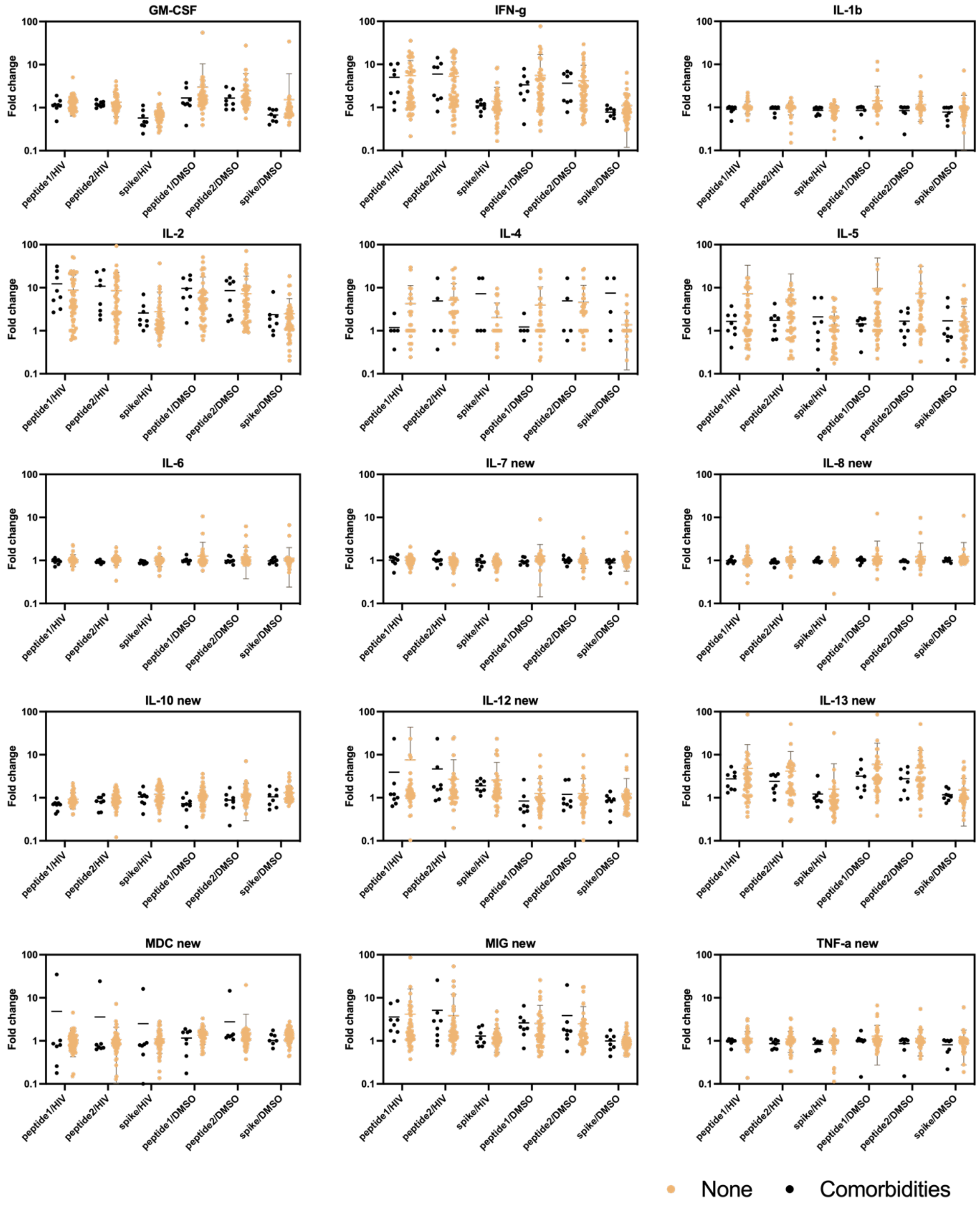

Figure S13 Dot graphs presenting protein secretion (as fold change) upon stimulation of PBMCs with SARS-CoV-2-derived peptides and Spike protein, relative to protein secretion upon DMSO or HIV peptide treatment, categorized by comorbidities.

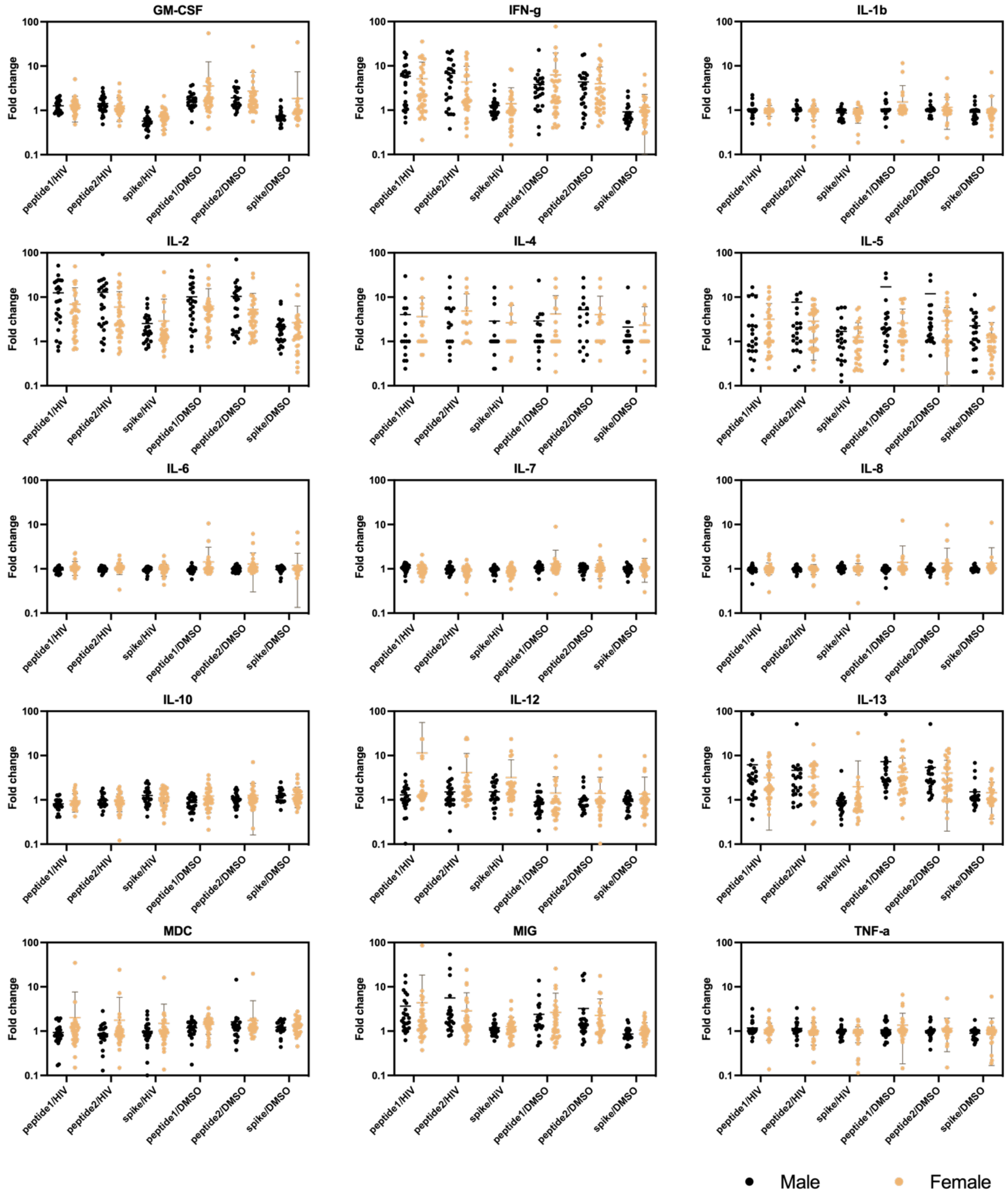

Figure S14 Dot graphs presenting protein secretion (as fold change) upon stimulation of PBMCs with SARS-CoV-2-derived peptides and Spike protein, relative to protein secretion upon DMSO or HIV peptide treatment, categorized by gender.

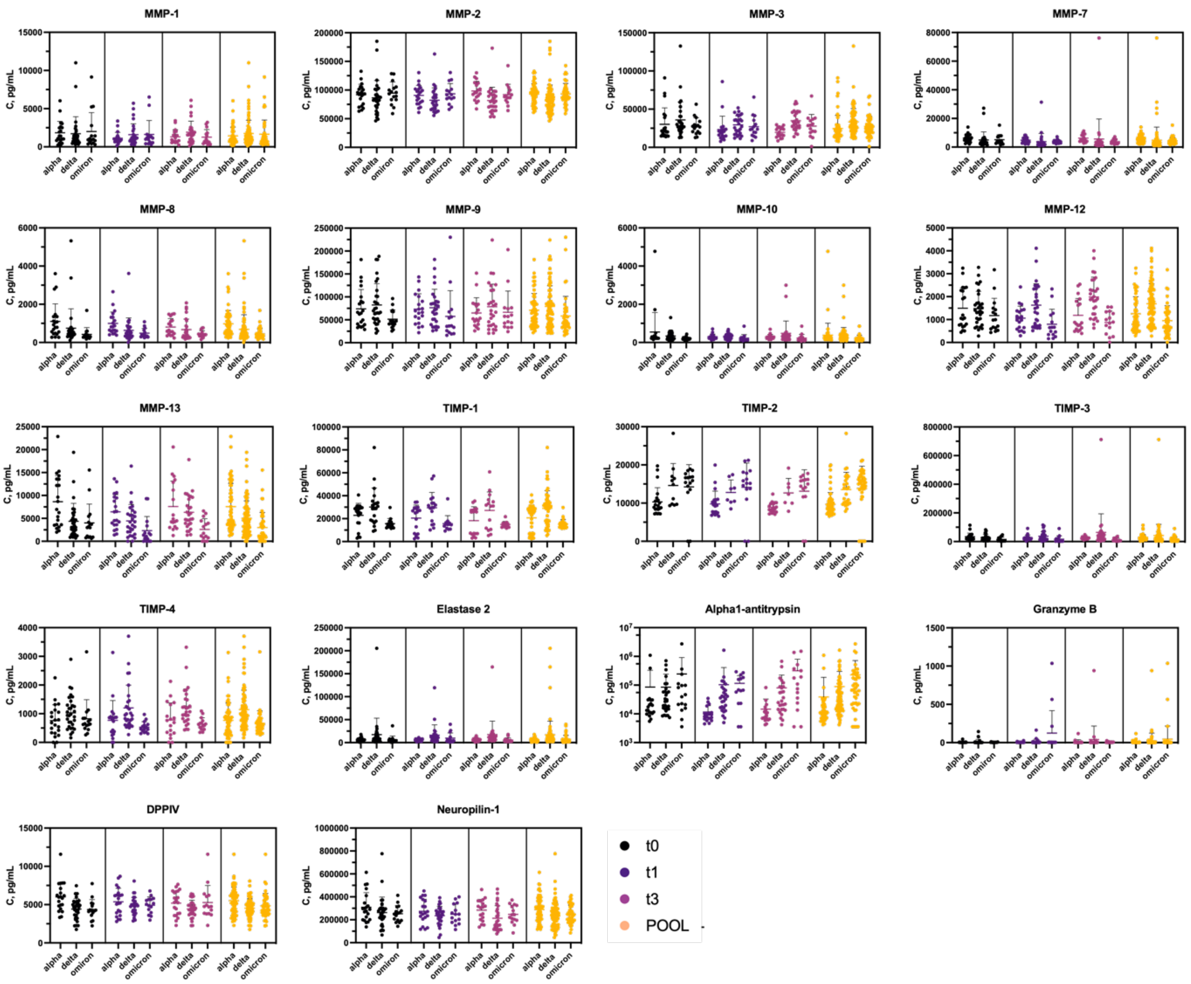

Figure S15 Nested dot graphs presenting the levels of selected proteases, their inhibitors, and other proteins (in pg/mL) at three time points (t0, t1, t3) in COVID-19 convalescent individuals, categorized by COVID-19 variant.

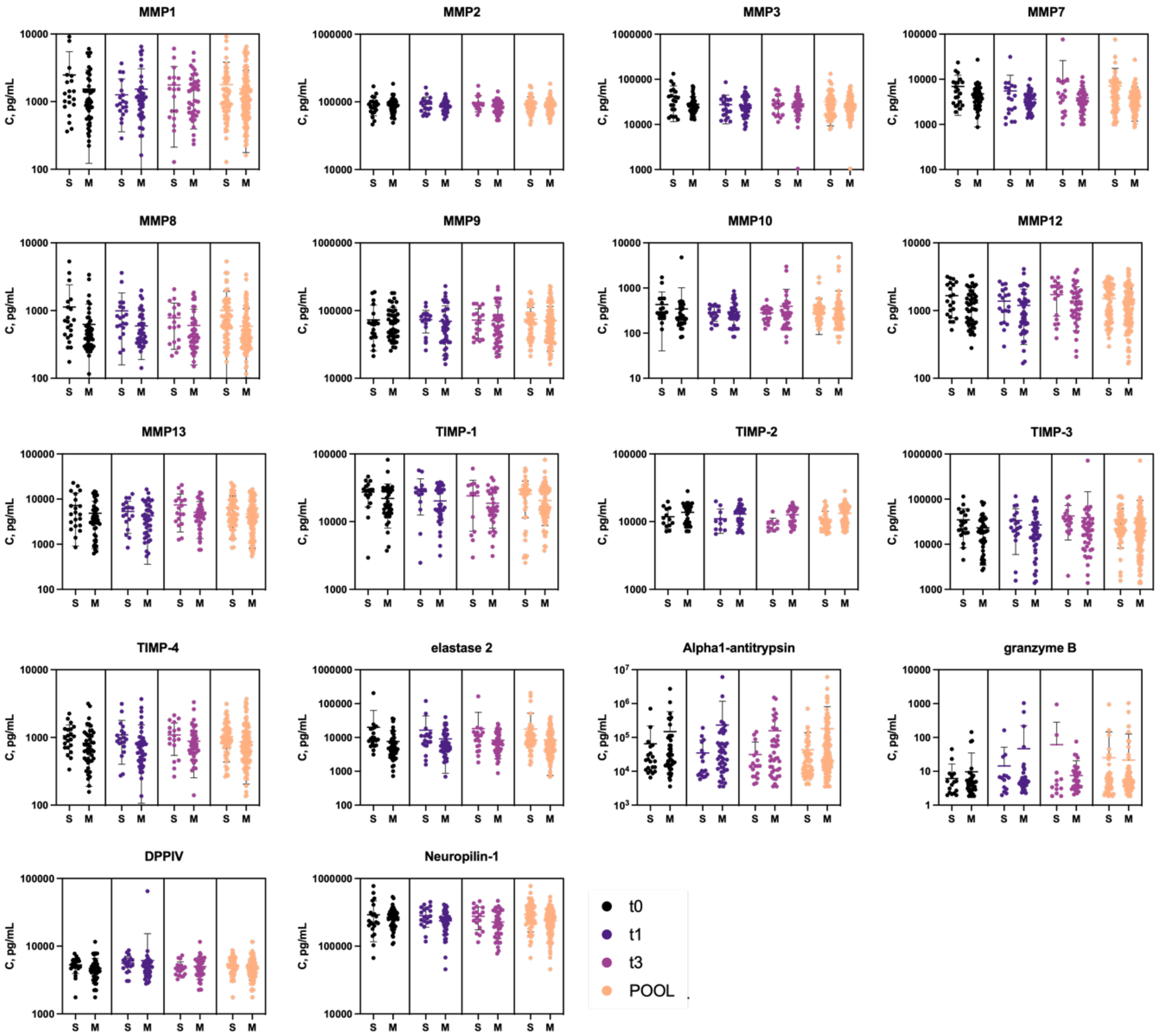

Figure S16 Nested dot graphs presenting the levels of selected proteases, their inhibitors, and other proteins (in pg/mL) at three time points (t0, t1, t3) in COVID-19 convalescent individuals, categorized by COVID-19 severity (mild/severe).

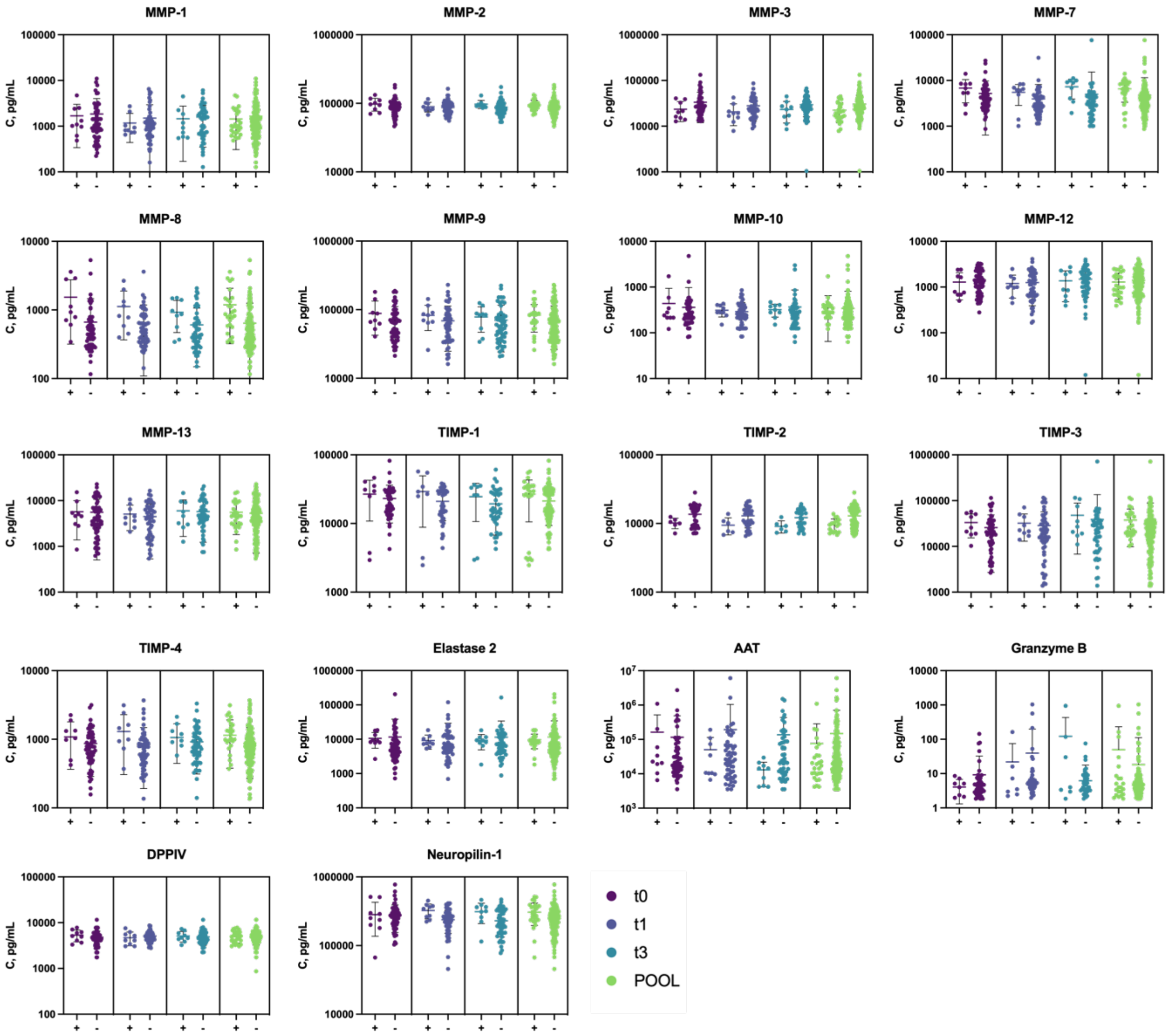

Figure S17 Nested dot graphs presenting the levels of selected proteases, their inhibitors, and other proteins (in pg/mL) at three time points (t0, t1, t3) in COVID-19 convalescent individuals, categorized by comorbidities.

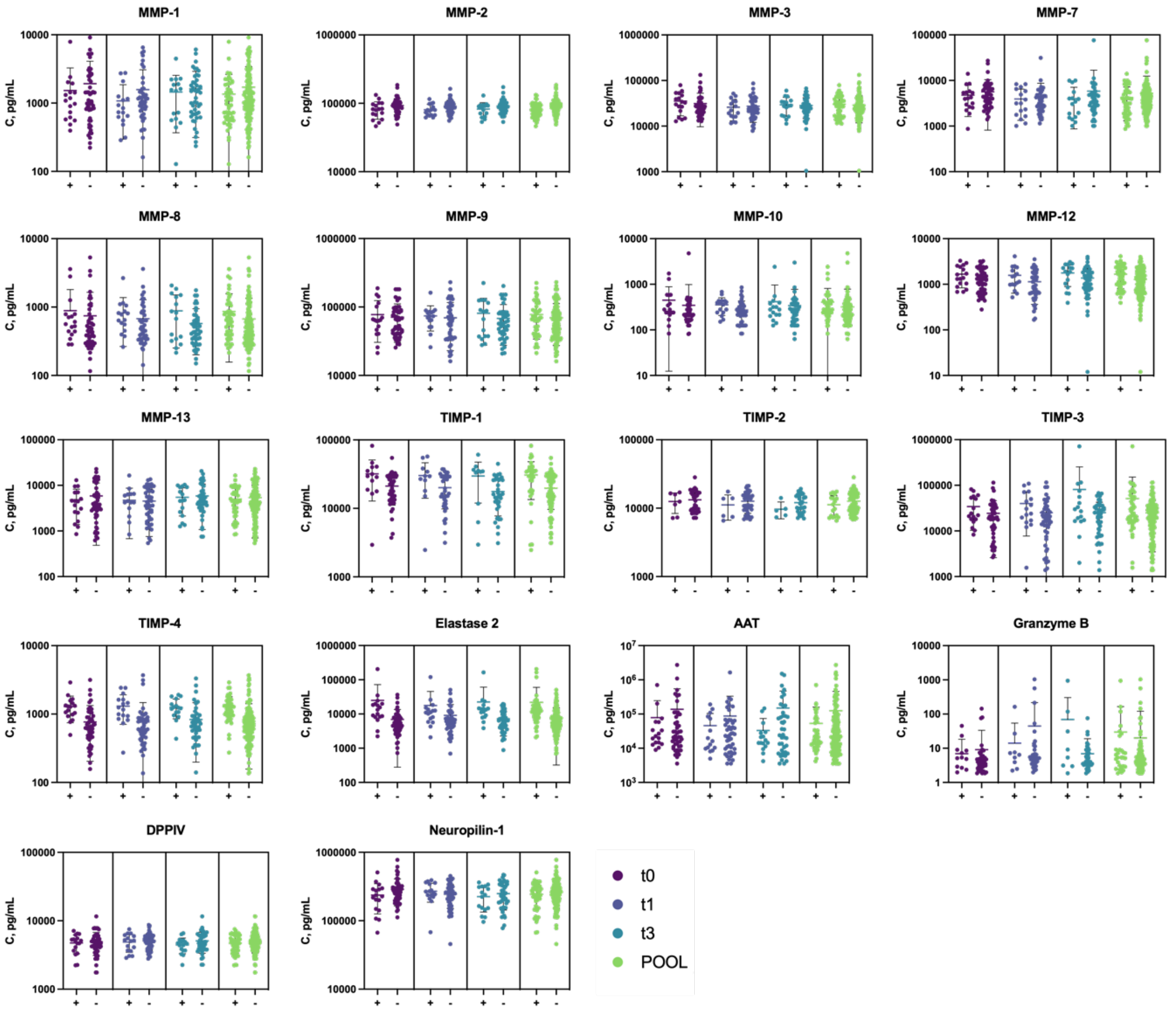

Figure S18 Nested dot graphs presenting the levels of selected proteases, their inhibitors, and other proteins (in pg/mL) at three time points (t0, t1, t3) in COVID-19 convalescent individuals, categorized by immunosuppressive treatment (+/-).

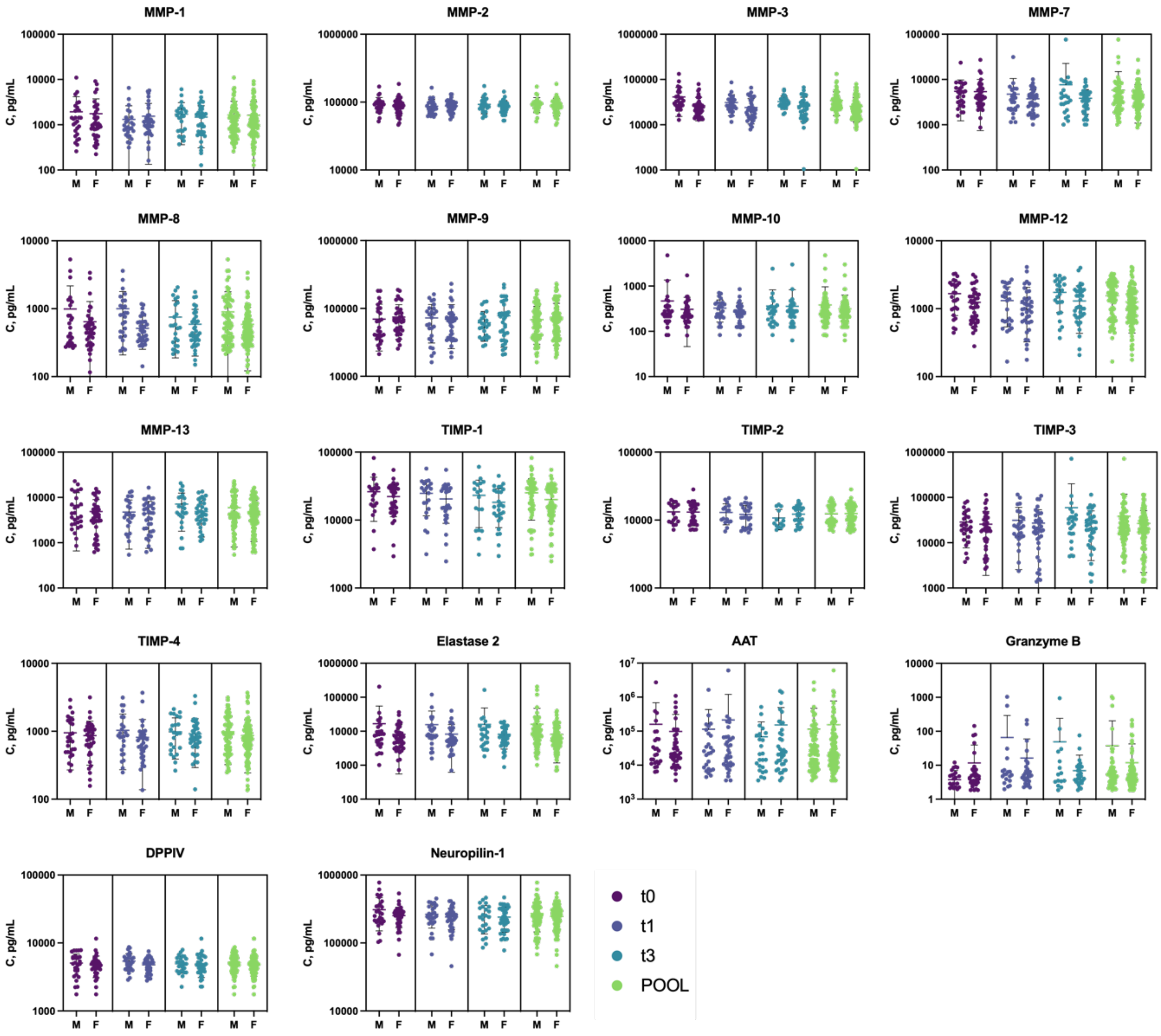

Figure S19 Nested dot graphs presenting the levels of selected proteases, their inhibitors, and other proteins (in pg/mL) at three time points (t0, t1, t3) in COVID-19 convalescent individuals, categorized by gender.

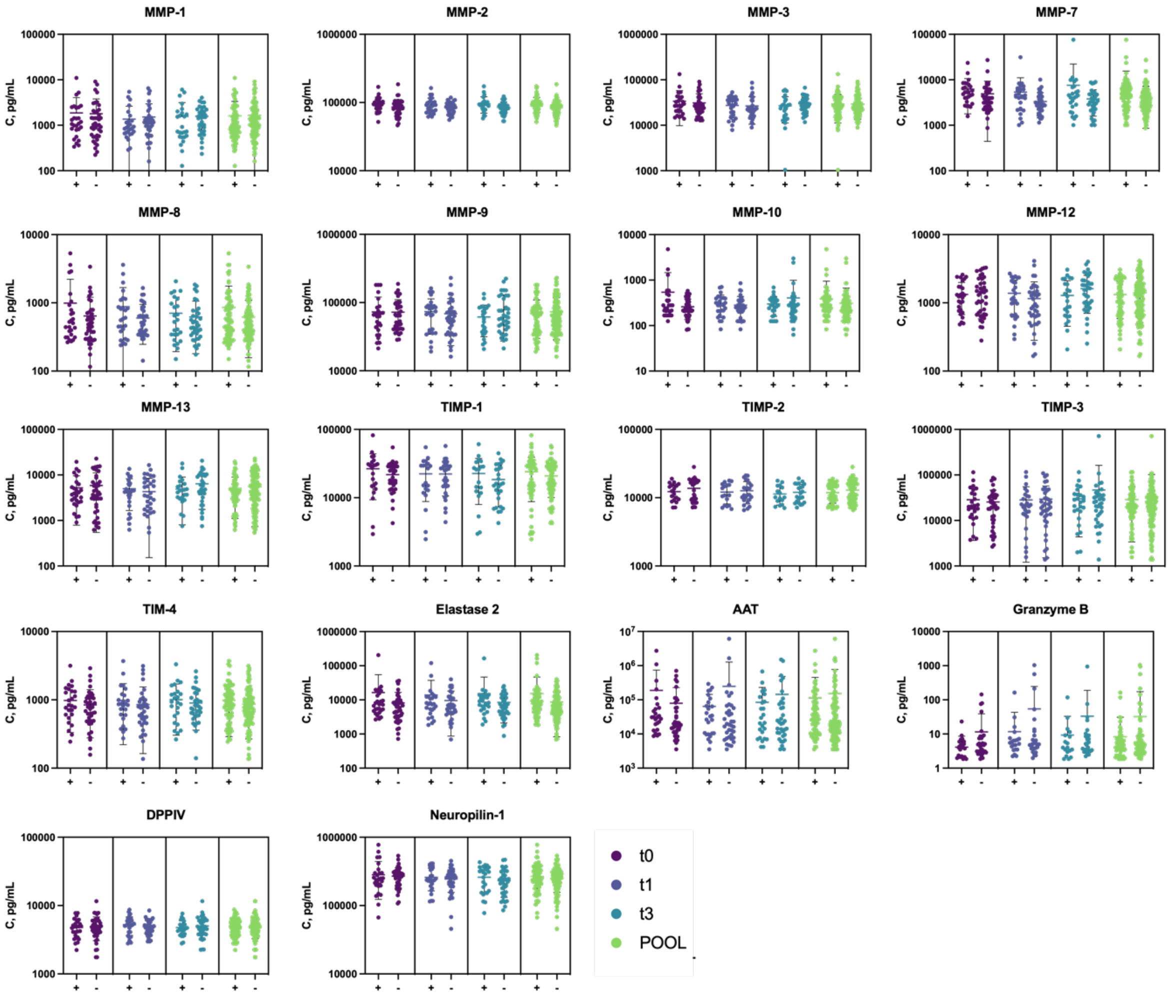

Figure S20 Nested dot graphs presenting the levels of selected proteases, their inhibitors, and other proteins (in pg/mL) at three time points (t0, t1, t3) in COVID-19 convalescent individuals, categorized by lung ultrasound results (- no changes, + negative changes).

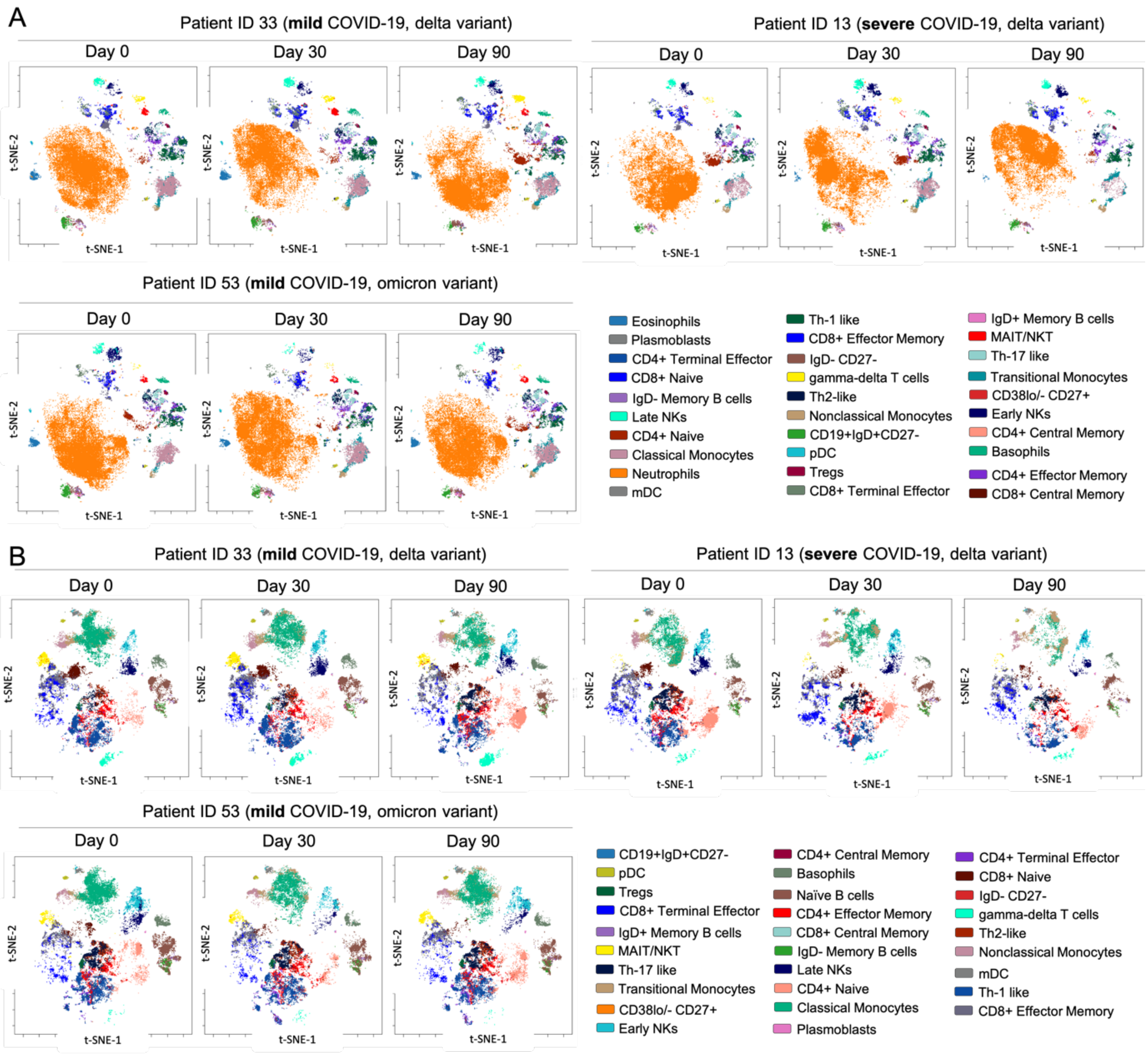

C

Patient ID	Age, years	Gender	COVID-19 severity	WHO Day 0	Lung ultrasound	Comorbidities
			**Alpha variant**			
2	61		severe	4	1	hypertension, asthma
7	69		mild	2	1	obesity, allergies
			**Delta** variant			
13	48	Male	severe	3	2,3	hypertension
33	46	Male	mild	2	3	none
			**Omicron** variant			
53	70	Female	mild	2	2,5	none

Figure S21 High-dimensional analysis of the immune landscape in COVID-19 convalescent patients by mass cytometry. **A** viSNE analysis of 30 immune cell subtypes in representative patients across Alpha, Delta, and Omicron COVID-19 variants. **B** viSNE analysis of 28 immune cell subtypes (lymphocytes, monocytes, and DCs; excluding polynuclear cells) in representative patients across Alpha, Delta, and Omicron COVID-19 variants. **C** Table summarizing some clinical information for patients selected for mass cytometry analysis.

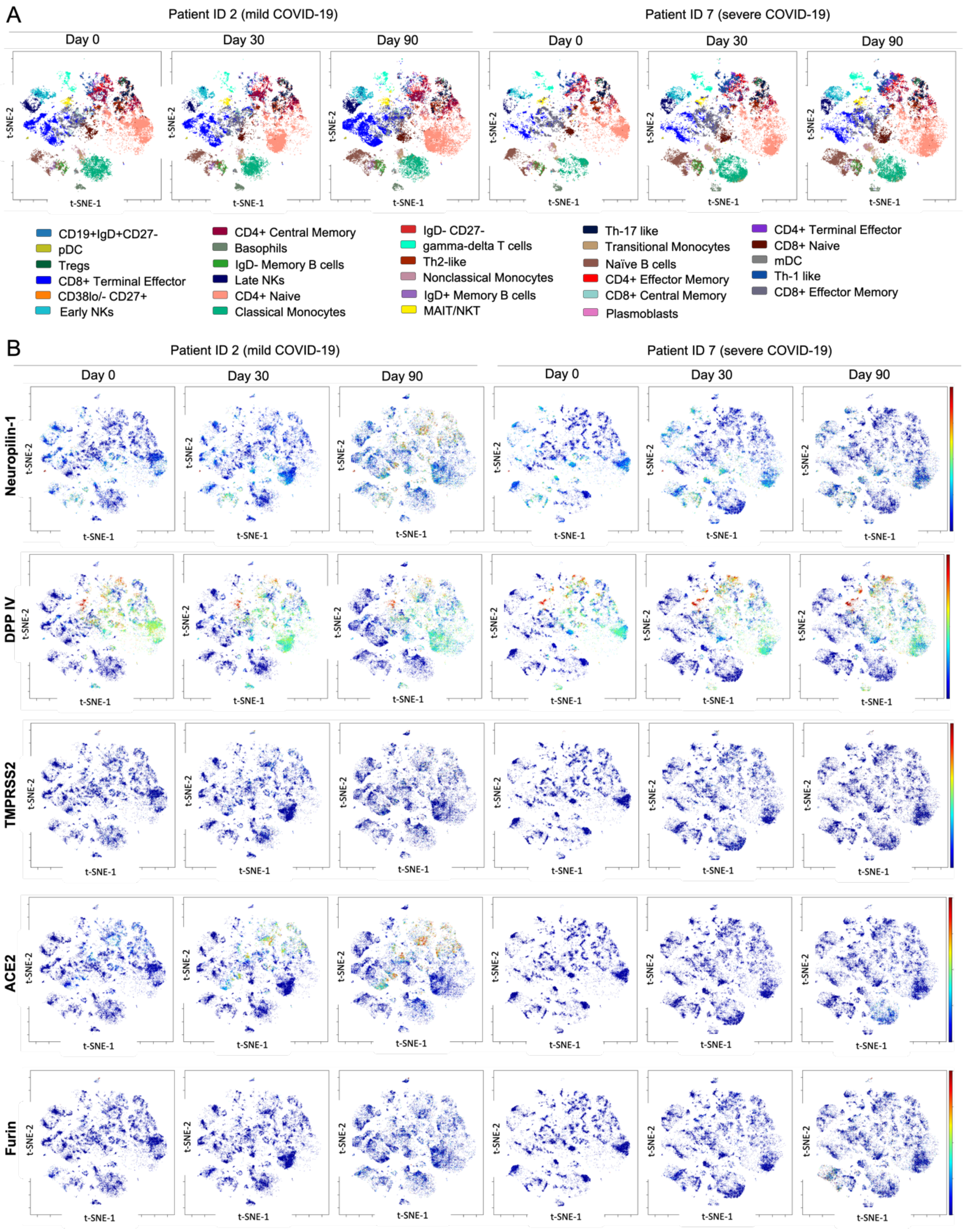

Figure S22 High-dimensional analysis of the immune landscape in COVID-19 convalescent patients by mass cytometry. (A) Analysis of immune signatures in monocytes, lymphocytes, and DCs presented as viSNE maps in two representative patients with mild and severe COVID-19. (B) viSNE maps presenting the expression and localization of COVID-19-related proteins: neuropilin-1, DPPIV, TMPRSS2, ACE2, and furin.

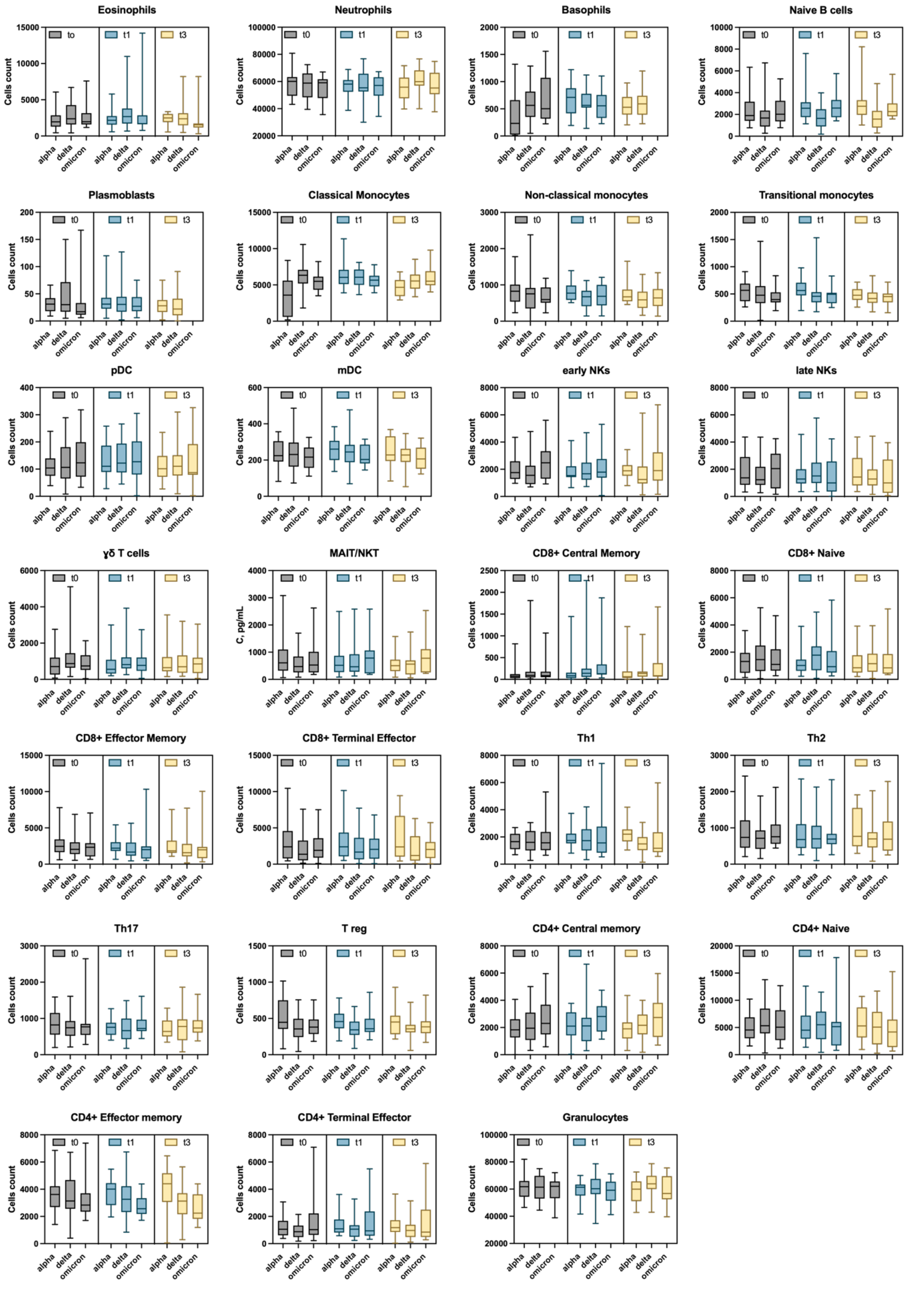

Figure S23 Nested box plots presenting the number of immune cells calculated per 100,000 cells, categorized by COVID-19 variant at three time points (t0, t1, t3).

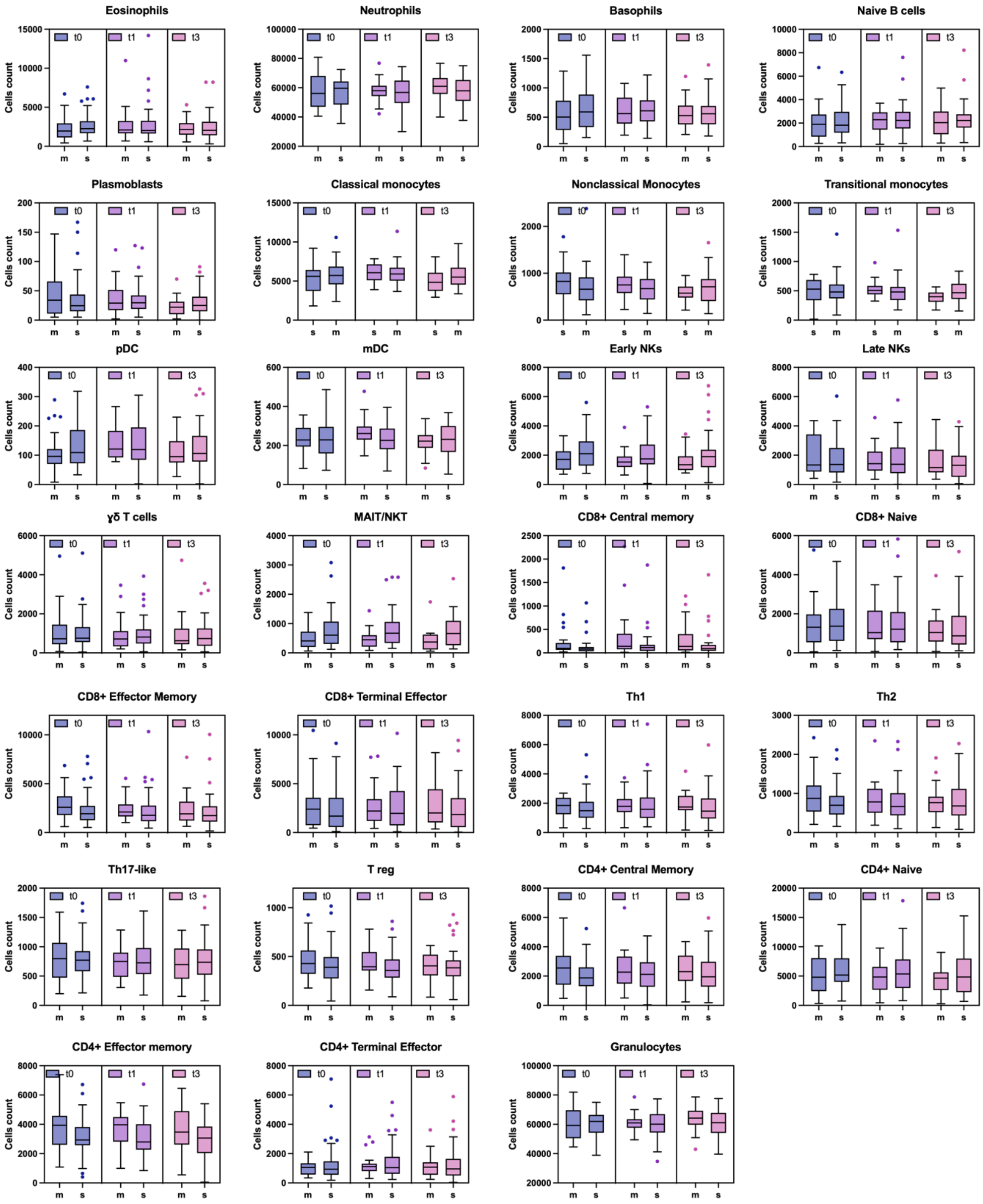

Figure S24 Nested box plots presenting the number of immune cells calculated per 100,000 cells, categorized by COVID-19 severity (m – mild, s – severe) at three time points (t0, t1, t3).

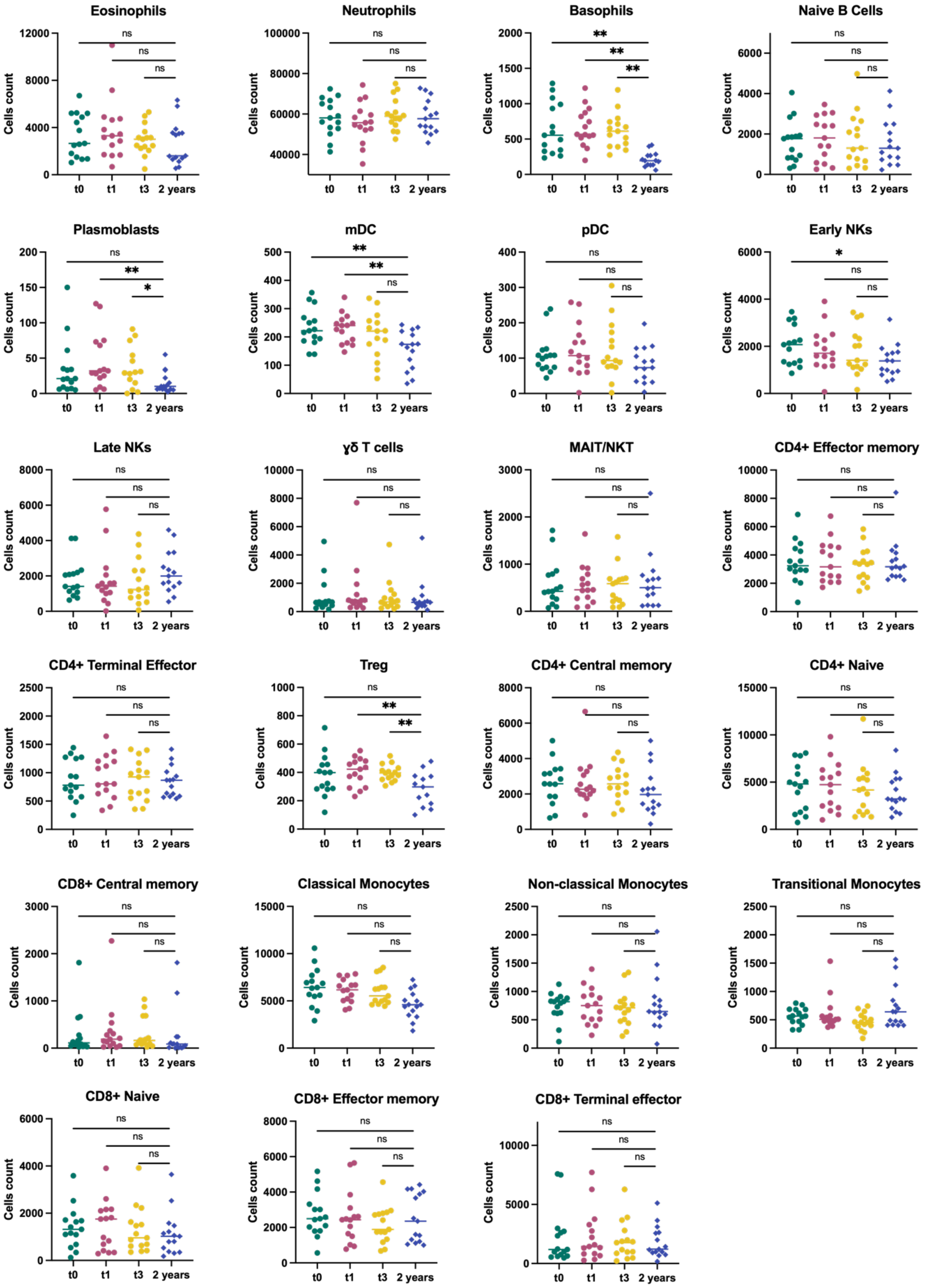

Figure S25 Nested box plots presenting the number of immune cells calculated per 100,000 cells in 15 patients during a 2-year follow-up study.

no.	kit name	catalog number	vendor
1	MILLIPLEX Human High Sensitivity T Cell Magnetic Bead Panel	HSTCMAG-28SK	Merck Group
2	MILLIPLEX Human MMP Magnetic Bead Panel 2	HMMP2MAG-55K	
3	MILLIPLEX Human MMP Magnetic Bead Panel 1	HMMP1MAG-55	
4	MILLIPLEX HUMAN CYTOKINE/CHEMOKINE/GROWTH FACTOR PANEL A Magnetic Bead Panel	HCYTA-60K	
5	MILLIPLEX HUMAN Sepsis Magnetic Bead Panel	HSP3MAG-63K	
6	MILLIPLEX HUMAN NEURODEGENERATIVE DISEASE Magnetic Bead Panel 2	HNDG2MAG-36K	
7	MILLIPLEX HUMAN TIMP Magnetic Bead Panel 2	HTMP2MAG-54K	
8	MILLIPLEX HUMAN Sepsis Panel 2 Magnetic Bead Panel	HSP2MAG-63K	
9	MILLIPLEX Human Cardiovascular Disease (CVD) Panel 6 Magnetic Bead Panel	HCVD6MAG-67K	
10	MILLIPLEX HUMAN Sepsis Panel 3 Magnetic Bead Panel	HSP3MAG-63K	
11	MILLIPLEX SARS-COV-2 Antigen Panel 1 IgG	HC19SERG1-85K	
12	MILLIPLEX SARS-CoV-2 Antigen Panel 1 IgM	HC19SERM1-85K	
13	MILLIPLEX Human Angiogenesis Panel 2 Magnetic Bead Panel	HANG2MAG-12K	

Table S1 A list of antibody panels used for Luminex analysis of antibody and protein levels in serum

no.	monoclonal antibody	clone	dilution	catalog number	vendor
1	Anti-Neuropilin-1	446921	1:370	MAB3870	R & D Systems
2	Anti-Furin	222722	1:370	MAB1503	
3	Anti-ACE-2	535919	1:310	MAB9332	
4	Anti-TMPRSS2	1038127	1:370	MAB10723	
5	Anti-DPPIV/CD26	222113	1:370	MAB1180	

Table S2 A list of *in house* conjugated metal-labelled antibodies used for immune profiling of COVID-19 convalescent patients by mass cytometry

no.	antibody	clone	vendor and catalog number
1	CD45	HI30	Standard Biotools 201325
2	CD196 (CCR6)	G034E3	
3	CD123	6H6	
4	CD19	HIB19	
5	CD4	RPA-T4	
6	CD8a	RPA-T8	
7	CD11c	Bu15	
8	CD16	3G8	
9	CD45RO	UCHL1	
10	CD45RA	HI100	
11	CD161	HP-3G10	
12	CD194 (CCR4)	L291H4	
13	CD25	BC96	
14	CD27	O323	
15	CD57	HNK-1	
16	CD183 (CXCR3)	G025H7	
17	CD185 (CXCR5)	J252D4	
18	CD28	CD28.2	
19	CD38	HB-7	
20	CD56 (NCAM)	NCAM16.2	
21	TCRγδ	B1	
22	CD294	BM16	
23	CD197 (CCR7)	G043H7	
24	CD14	63D3	
25	CD3	UCHT1	
26	CD20	2H7	
27	CD66b	G10F5	
28	HLA-DR	LN3	
29	IgD	IA6-2	
30	CD127	A019D5	

Table S3 A list of metal-labelled antibodies comprising Maxpar® Direct™ Immune Profiling Assay™ used for the immunological analysis of whole blood samples from COVID-19 convalescent patients, by mass cytometry.
